# The gait pattern and not the femoral morphology is the main contributor to asymmetric hip joint loading

**DOI:** 10.1371/journal.pone.0291789

**Published:** 2023-09-26

**Authors:** Willi Koller, Arnold Baca, Hans Kainz

**Affiliations:** 1 Department of Biomechanics, Kinesiology and Computer Science in Sport, Centre for Sport Science and University Sports, University of Vienna, Vienna, Austria; 2 Neuromechanics Research Group, Centre for Sport Science and University Sports, University of Vienna, Vienna, Austria; 3 Vienna Doctoral School of Pharmaceutical, Nutritional and Sport Sciences, University of Vienna, Vienna, Austria; Polytechnic Institute of Setúbal: Instituto Politecnico de Setubal, PORTUGAL

## Abstract

Gait asymmetry and skeletal deformities are common in many children with cerebral palsy (CP). Changes of the hip joint loading, i.e. hip joint contact force (HJCF), can lead to pathological femoral growth. A child’s gait pattern and femoral morphology affect HJCFs. The twofold aim of this study was to (1) evaluate if the asymmetry in HJCFs is higher in children with CP compared to typically developing (TD) children and (2) identify if the bony morphology or the subject-specific gait pattern is the main contributor to asymmetric HJCFs. Magnetic resonance images (MRI) and three-dimensional gait analysis data of twelve children with CP and fifteen TD children were used to create subject-specific musculoskeletal models and calculate HJCF using OpenSim. Root-mean-square-differences between left and right HJCF magnitude and orientation were computed and compared between participant groups (CP versus TD). Additionally, the influence on HJCF asymmetries solely due to the femoral morphology and solely due to the gait pattern was quantified. Our findings demonstrate that the gait pattern is the main contributor to asymmetric HJCFs in CP and TD children. Children with CP have higher HJCF asymmetries which is probably the result of larger asymmetries in their gait pattern compared to TD children. The gained insights from our study highlight that clinical interventions should focus on normalizing the gait pattern and therefore the hip joint loading to avoid the development of femoral deformities.

## Introduction

Gait asymmetry [1] and skeletal deformities [2–4] are common in many children with cerebral palsy (CP). Children with CP are born with typical bony geometry, but in many patients the femoral neck shaft angle (NSA) and anteversion angle (AVA) does not decrease during growth as in typically developing (TD) children [2, 5, 6]. In TD children the NSA and the AVA approximately decrease from 150° to 120° [7] and from 40° to 15° [2, 8] during childhood, respectively. The pathologic femoral geometry in children with CP affects moment arms of muscles [9], which might have an impact on the patient’s gait pattern [10]. CP gait differs widely between patients and can be classified based on the joint kinematics of the lower limbs e.g. equinus drop foot gait, jump gait or crouch gait [11]. Gait asymmetries are higher in patient with hemiplegic CP compared to patients with diplegic CP. However, many children with diplegic CP have a more affected leg, which can also lead to an asymmetric gait pattern [1].

Bone is adaptive to mechanical loading [12, 13] and the hip joint contact force (HJCF) is one of the main biomarkers, which determines femoral bone growth [14–16]. Musculoskeletal simulations can be used to estimate subject-specific HJCF [17]. In a recent simulation study, Kainz et al. [18] showed that the orientation of the HJCF in the sagittal plane can differentiate between children with CP who are likely to have typical and pathological femoral growth. Asymmetric HJCF can alter growth plate loading [19] and therefore lead to asymmetric development of bones in length and shape resulting in altered biomechanics. Previous studies showed that not only the subject-specific gait pattern but also the femoral geometry has a big impact on the estimation of the HJCF [16, 20–22].

Single event multi-level surgeries, including de-rotation osteotomies, are frequently performed in children with CP to address femoral deformities, enhance their gait pattern, hinder the progression of additional impairments and normalize joint loadings [23–25]. The femoral geometry and the gait pattern interrelate with each other, i.e. the geometry influences muscle moment arms, altered moment arms might change muscle forces and/or the gait pattern resulting in altered loading which subsequently modifies the femoral growth and geometry. Considering that both, the gait pattern and femoral geometry, influence joint loads, it is difficult to assess any cause-effect relationships with traditional experimental studies. Hence, it is not known if the child’s gait pattern or the subject-specific femoral geometry is the main contributor to asymmetric HJCFs in children with CP. Furthermore, it is unknown if the asymmetry in HJCFs is higher in children with CP compared to TD children.

The twofold aim of this study was to (1) evaluate if the asymmetry in HJCFs is higher in children with CP compared to TD children and (2) identify, if the bony femoral morphology or the subject-specific gait pattern is more associated with asymmetries of HJCFs. We conducted what-if simulations to identify whether normalizing the gait pattern or correcting the bony geometry is of utmost importance in clinical interventions to normalize joint loadings. Considering that CP is a disease where both legs can be affected by different severity leading to gait variations and induced compensation mechanisms on the less affected leg, we hypothesized that the asymmetry in hip loading, i.e. HJCF magnitude and orientation, is higher in children with CP compared to TD children. Furthermore, we assumed that the gait pattern has a bigger impact on asymmetries in HJCF than the subject-specific femoral geometry because the gait pattern alters ground reaction forces and therefore joint moments at each joint whereas the femoral geometry, i.e. NSA and AVA, only alters the moment arms of a small number of muscles.

## Methods

Magnetic resonance imaging (MRI) data and three-dimensional gait analysis (3DGA) data including marker trajectories and ground reaction forces of twelve children diagnosed with CP (10.4±3.7 years old, height: 133.6±14.9 cm, mass: 30.1±10.1 kg) and fifteen TD children (10.3±2.6 years old, height: 146.3±11.9 cm, mass: 40.1±14.8 kg) were analyzed for this study. The data of all CP children and five TD children was captured during a previous study [26] while the data of the remaining ten TD children was additionally collected for the purpose of this study. The sample of the newly recorded dataset was planned to match the age of the CP cohort and to ensure a comparable number of participants in the TD and CP groups. Walking without an assistant device in daily life was the main inclusion criteria for participants with CP. Three and nine of these children were classified as level 1 and 2 based on the Gross Motor Function Classification System (GMFCS), respectively. Five and seven of the participants were diagnosed with hemi- and diplegic CP, respectively. Our participants with CP walked with a variety of pathological gait patterns including true equinus, equinus jump gait, apparent equinus and crouch gait. Further details are provided in S1 Table in the S1 File. None of the TD participants had any previous major injuries, surgeries or pain at the lower limbs. Ethics approval was obtained from the local ethics committees (University of Vienna, reference number 00578). Written informed consent to participate in this study was provided by the participants’ legal guardian.

### Data collection

Data collection of the retrospective analyzed data (CP children and 5 TD children) is described in detail in Kainz et al. [26]. In short, MRIs of the pelvis and lower limbs were collected using a 1.5 Tesla MRI scanner (MAGNETOM Avanto, Siemens, Berlin/Munich, Germany) with a voxel size of 1.1x1.1x1.1 mm. Motion capture data were collected using an 8-camera 3D motion capture system (Vicon Motion Systems, Oxford, UK) with an extended Plug-in-Gait marker set with additional clusters of three markers on each thigh and shank segment and an additional marker at the 5th metatarsal head of each foot [27–29]. MRI images of the additionally recorded data (ten TD children) were collected using a 3T magnetic resonance scanner (MAGNETOM Vida, Siemens, Berlin/Munich, Germany) with a T1 vibe sequence with a voxel size of 0.8x0.8x0.7 mm. 3DGA-data for these ten TD children were captured on the same day as the MRI images using a 12 camera motion capture system (Vicon Motion Systems, Oxford, UK) with a camera sampling frequency of 200 Hz. The used marker set during the motion capturing was the same as in the retrospective dataset. Simultaneously, ground reaction forces were acquired with 1000 Hz using five force plates (Kistler Instrumente, Winterthur, Switzerland). All children performed several gait trials with a self-selected walking speed. Marker trajectories were captured, labelled, and filtered (Butterworth 4^th^order, 6Hz low-pass filter) in Nexus 2.12.1 (Vicon Motion System, Oxford, UK). The retrospective and prospective datasets included the same types of experimental data and all further simulations and analyses were performed with the same workflow for both datasets.

### Segmentation of MRIs

All MRIs were segmented using 3D Slicer [30] and each femur was exported as a STL-file. The STL mesh was subsequently used to compute the NSA and AVA with a customized MATLAB (Mathworks Inc., Natick, MA, USA) script, described in detail in the supplementary material of Kainz et al. [16].

### Musculoskeletal models

**Fig 1:**
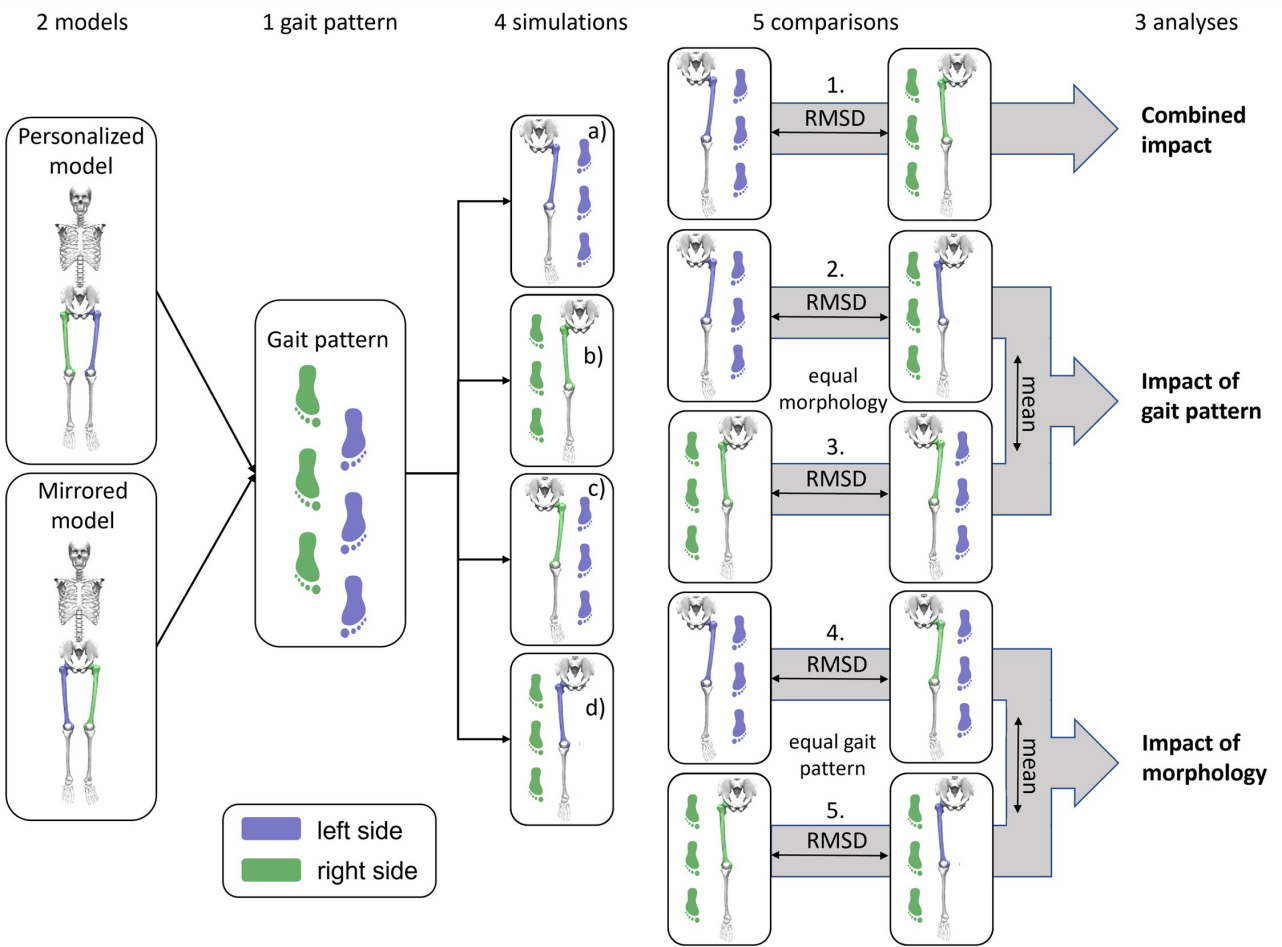
Combination of models and input data for four different simulations (a–d) and five comparisons (1.-5.) to identify the influence on the asymmetry of HJCF magnitude and its orientation solely due to the participants’ gait pattern (mean of comparison 2. and 3.), solely due to the participants morphology (mean of 4. and 5.) and the combination of both factors (comparison 1.).

The generic ‘gait2392’ OpenSim model [31] with locked metatarsophalangeal joints was used as the base model for the subject-specific models. The recently developed Torsion Tool [32] was used to personalize the femoral geometry of each model to match the child’s NSA and AVA (personalized model). One additional model (mirrored model) was created for each participant where the models’ femoral morphology of the right femur was modified according to the NSA and AVA of the participants’ left femur and vice versa. This allowed us to calculate the impact on the asymmetry solely due to the gait pattern and solely due to the femoral morphology, separately (Fig 1).

The personalized models were scaled to the participants anthropometry based on calculated joint centers (hip: equation from Sangeux [33]; knee: midpoint between medial and lateral knee markers; ankle: equation from Bruening [34]) and the location of surface markers [26]. Each leg was scaled independently to account for potential length discrepancies between the left and right leg. The mirrored model was scaled with the same scale settings. Hence, we had two models, which were exactly the same except for the femoral morphology and the corresponding muscle paths. The maximum isometric muscle forces of all models were scaled by Eq 1 to improve muscle force estimations [35, 36].


$$F_{scaled\;}=F_{generic\;}\;*\;{\left(m_{scaled\;}/m_{generic\;}\right)}^{\left(2/3\right)}$$ (1)

### Musculoskeletal simulations

All models and the corresponding gait analysis data were used to calculate joint angles, joint moments, muscle forces and joint contact forces using inverse kinematics, inverse dynamics, static optimization (minimizing the sum of squared muscle activations) and joint reaction load analyzes [37], respectively. Knee and ankle joint markers were only used for scaling and excluded during inverse kinematics. The remaining markers were weighted equally. Maximum marker errors and root-mean-square errors were accepted if less than 4 cm and 2 cm, respectively, as recommended by OpenSim’s best practice recommendations [38]. On average the mean waveforms of 8.4±4.1 steps (minimum of 3 steps for each side) were computed for each parameter and further analyzed. All simulation were performed with MATLAB R2021a and OpenSim 4.2 [39].

### Data analysis

The intra-subject differences of the NSA (∆NSA) and AVA (∆AVA) were calculated as the absolute difference between the values of the left and right side (Eqs 2 and 3). A score to quantify the asymmetry of the intra-subject femoral morphology was calculated by the sum of the differences in AVA and NSA between the left and right femur (Eq 4) and is further referred to as morphology asymmetry score (MAS).


$$∆NSA=|NSA_{left\;}-NSA_{right\;}|$$ (2)

$$∆AVA=|AVA_{left\;}-AVA_{right\;}|$$ (3)

$$MAS=|AVA_{left\;}-AVA_{right\;}|+|NSA_{left\;}-NSA_{right\;}|$$ (4)

Walking speed was calculated, normalized to participants’ leg lengths [40] and compared between both groups. A gait asymmetry score (GAS) was used to quantify differences in joint angles between left and right leg of each participant. The GAS was calculated similar to the gait profile score [41] except that we compared joint angles between each participant’s right and left leg and not between a patient and a healthy control group.

All results of the musculoskeletal simulations were time normalized to the stance phase. Joint kinematics were reported according to the International Society for Biomechanics’ (ISB) recommendations [42]. HJCF magnitudes and orientations acting on the femur were calculated in the sagittal, transversal and frontal plane of the femurs’ coordinate system [43] and then compared between each participant’s left and right side. Performing the musculoskeletal simulations for the personalized and the mirrored model resulted in the following four simulations for each participant:

- left kinematics and left femoral morphology (left steps of personalized model)
- right kinematics and right femoral morphology (right steps of personalized model)
- left kinematics and right femoral morphology (left steps of mirrored model)
- right kinematics and left femoral morphology (right steps of mirrored model)

Subsequently, for each participant HJCF asymmetries were quantified by calculating root-mean-square-difference (RMSD) between the extracted waveforms (HJCF magnitude and orientation during stance phase) of different simulations. The following five comparisons between the different simulations were used to quantify HJCF asymmetries and the impact of each child’s gait pattern and femoral morphology on HJCF asymmetries (Fig 1):

1. RMSD of simulations a) and b) to identify the combined impact of morphology and gait pattern on HJCF asymmetries
2. RMSD of simulations a) and d) to identify the impact of the gait pattern on HJCF asymmetries
3. RMSD of simulations b) and c) to identify the impact of the gait pattern on HJCF asymmetries
4. RMSD of simulations a) and c) to identify the impact of the morphology on HJCF asymmetries
5. RMSD of simulations b) and d) to identify the impact of the morphology on HJCF asymmetries

### Statistics

One-sided independent samples t-tests were used to compare the NSA, AVA, the intra-subject difference of NSA (∆NSA) and AVA (∆AVA) and MAS between the CP and TD group.

Two between-within 2x3x2 ANOVAs were performed with SPSS Statistics 28.0 (IBM, New York, USA) to answer our research questions, i.e. identify significant differences in HJCF asymmetries (i) between CP and TD children and (ii) caused by the morphology versus the gait pattern. The cohort (CP versus TD) and the contribution (due to morphology versus due to gait pattern) were independent variables and the component/angle in 3 directions/planes of the HJCF were dependent variables. For pairwise comparisons, post-hoc Bonferroni correction was applied and Greenhouse-Geisser corrected values were used, if sphericity was violated. Additional independent t-tests were used to compare the combined influence of the morphology and the gait pattern on HJCF asymmetries between CP and TD children. For all tests, the significance level was set to p<0.05. Post-hoc power analyses were performed with GPower 3.1.9.7 [44] to quantify the statistical power of our main findings related to our research questions.

Furthermore, we evaluated if there are significant linear regression correlations between the asymmetry in femoral morphology (∆NSA, ∆AVA and MAS) and the asymmetry of HJCF magnitude and/or orientation. Results and discussion of these additional analysis can be found in the S1 File.

## Results

### Femoral morphology

**Fig 2:**
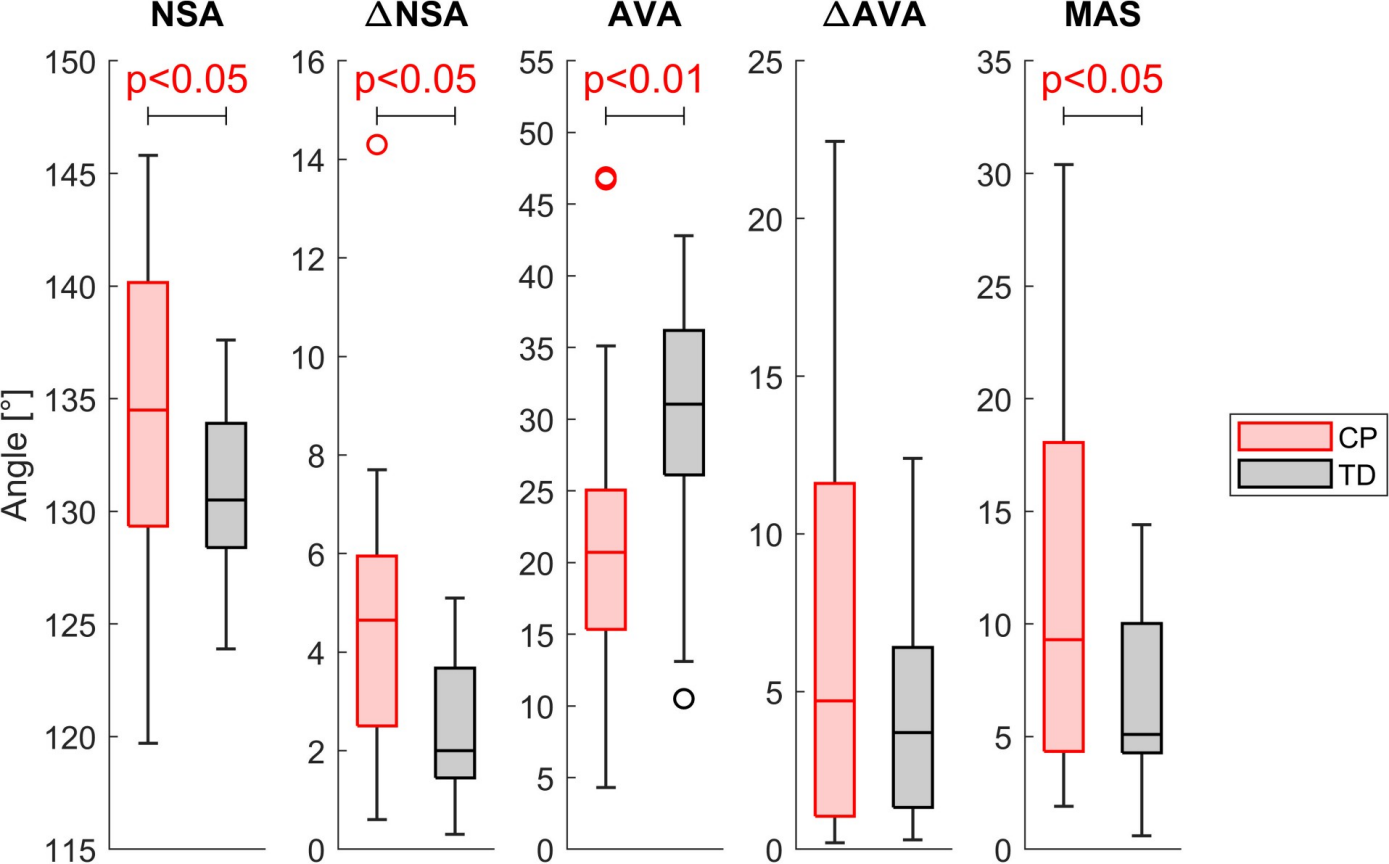
Comparison of the NSA, the AVA, the corresponding asymmetries (∆NSA and ∆AVA) and the MAS between the CP and the TD group.

The NSA (134.3±6.9°) and ∆NSA (4.9±3.5°) of the CP group were significantly higher (p<0.05) than the values of the TD group (NSA 130.9±3.5°, ∆NSA 2.6±1.5°). The AVA was significantly higher (p<0.01) in TD (29.7±8.8°) compared to CP children (22.3±10.2°), but ∆AVA did not differ between both groups. The MAS was significantly lower (p<0.05) in TD participants (7.0±4.1°) compared to participants with CP (11.9±9.1°) (Fig 2).

### Gait pattern and HJCF

**Fig 3:**
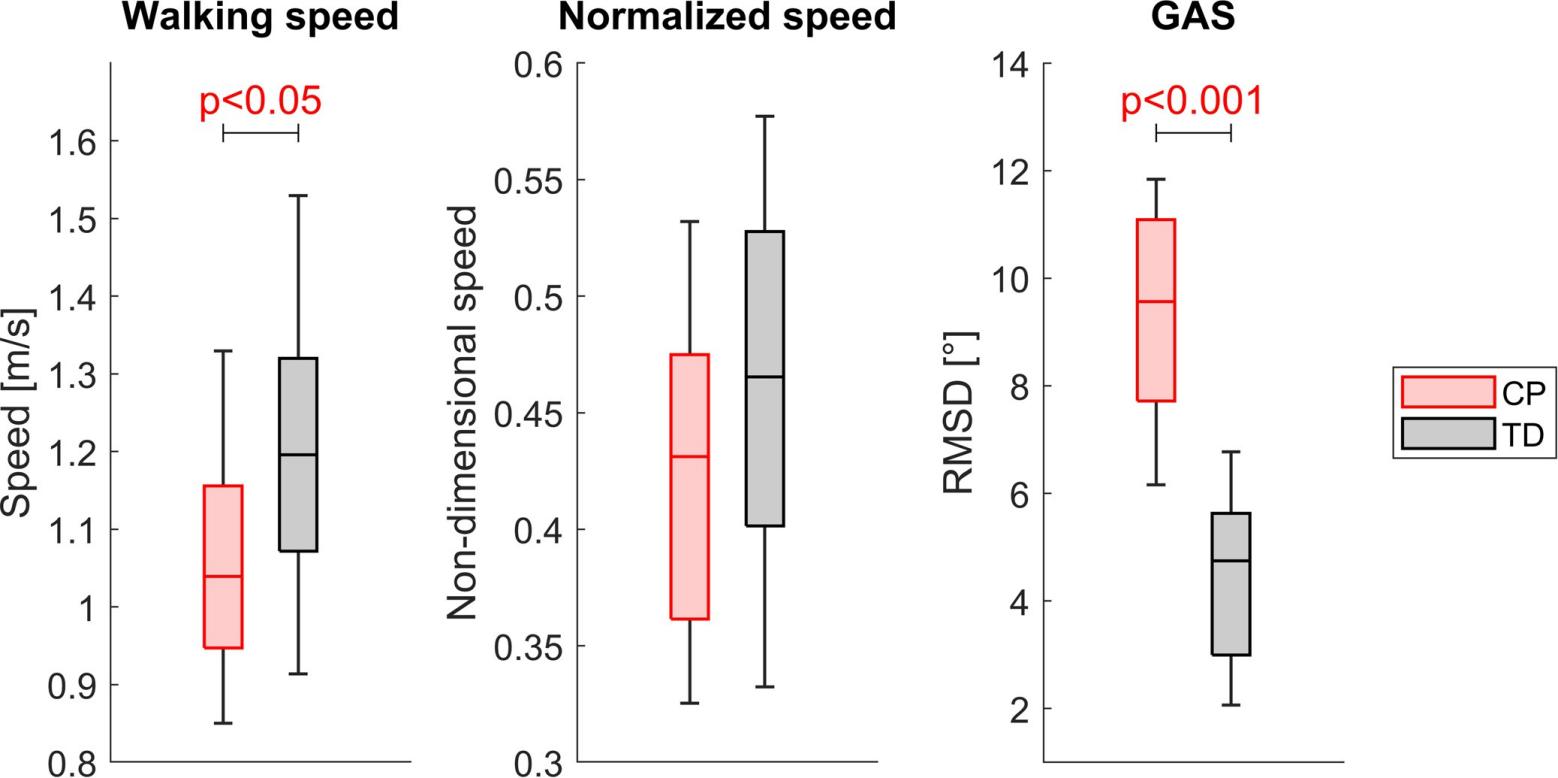
Comparison of the walking speed and the GAS between the CP and the TD group.

**Fig 4:**
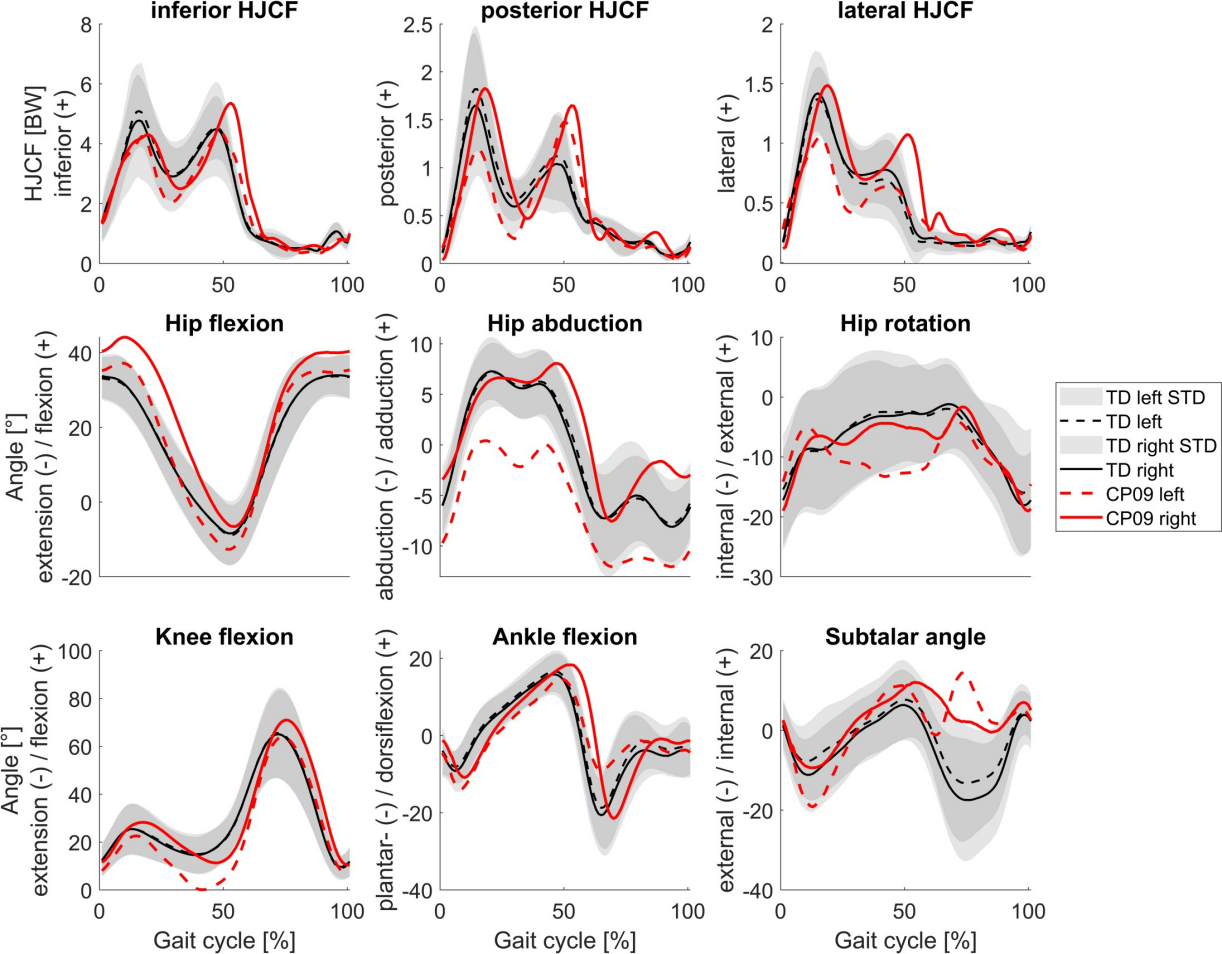
Mean HJCF represented in femurs’ coordinate system and joint angles during the gait cycle presented for TD children and HJCF and joint angles of a representative child with CP. CP01 was the participant with the lowest GAS. Figures with the gait kinematic waveforms of each participant with CP are included in the S1 File.

**Fig 5:**
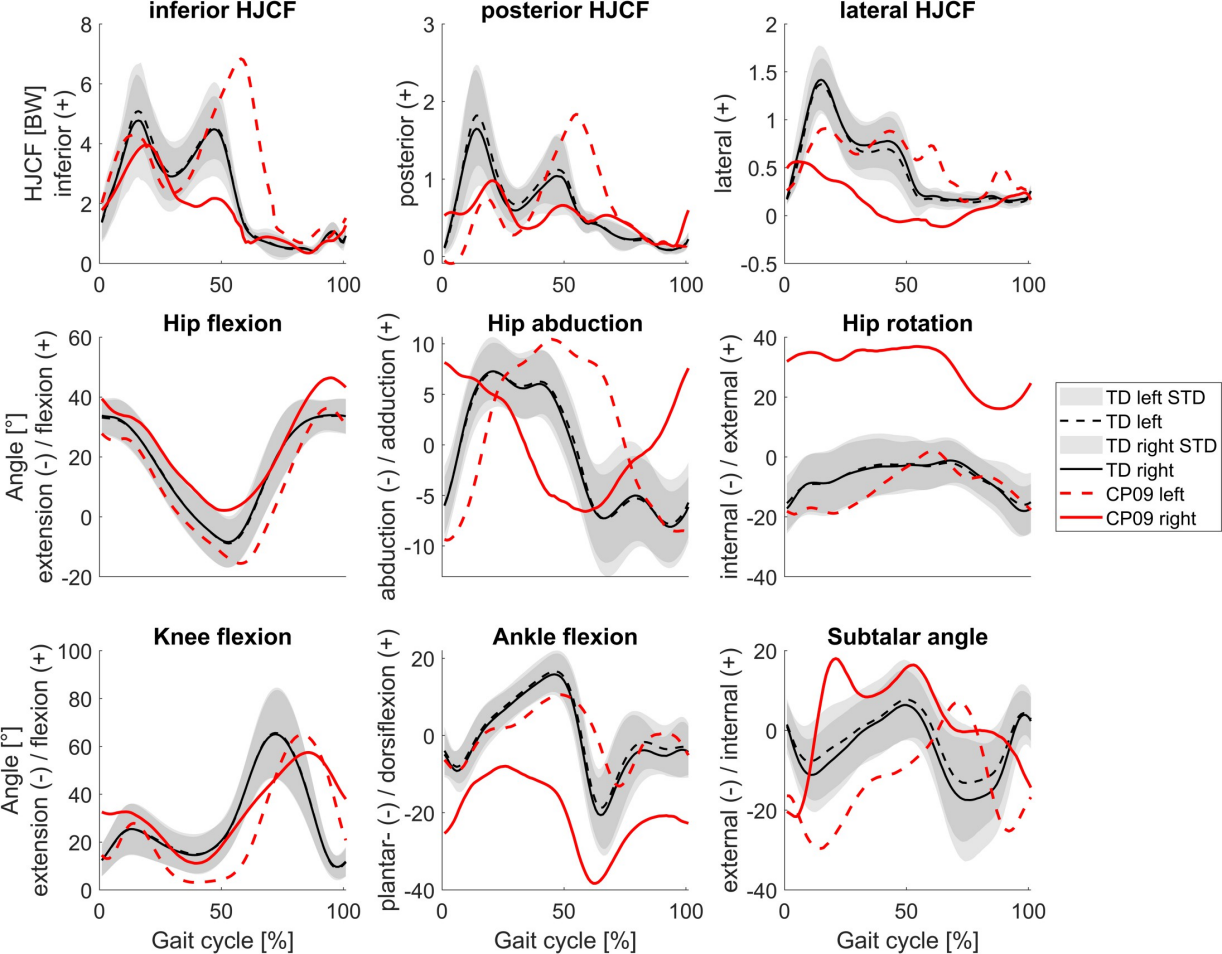
Mean HJCF represented in femurs’ coordinate system and joint angles during the gait cycle presented for TD children and HJCF and joint angles of a representative child with CP. CP09 was the participant with the highest GAS. Figures with the gait kinematic waveforms of each participant with CP are included in the S1 File.

The CP group walked significantly slower (p<0.01) than the TD group but walking speed normalized to leg length was not significantly different between both groups (Fig 3). Joint angles and HJCF during the gait cycle of TD participants were similar between both legs, whereas in some children with CP joint angles as well as HJCF differed vastly between the left and right side (Figs 4 and 5). The GAS was significantly higher (p<0.001) in CP (10.4±4.7°) compared to TD participants (4.5±1.5°).

**Fig 6:**
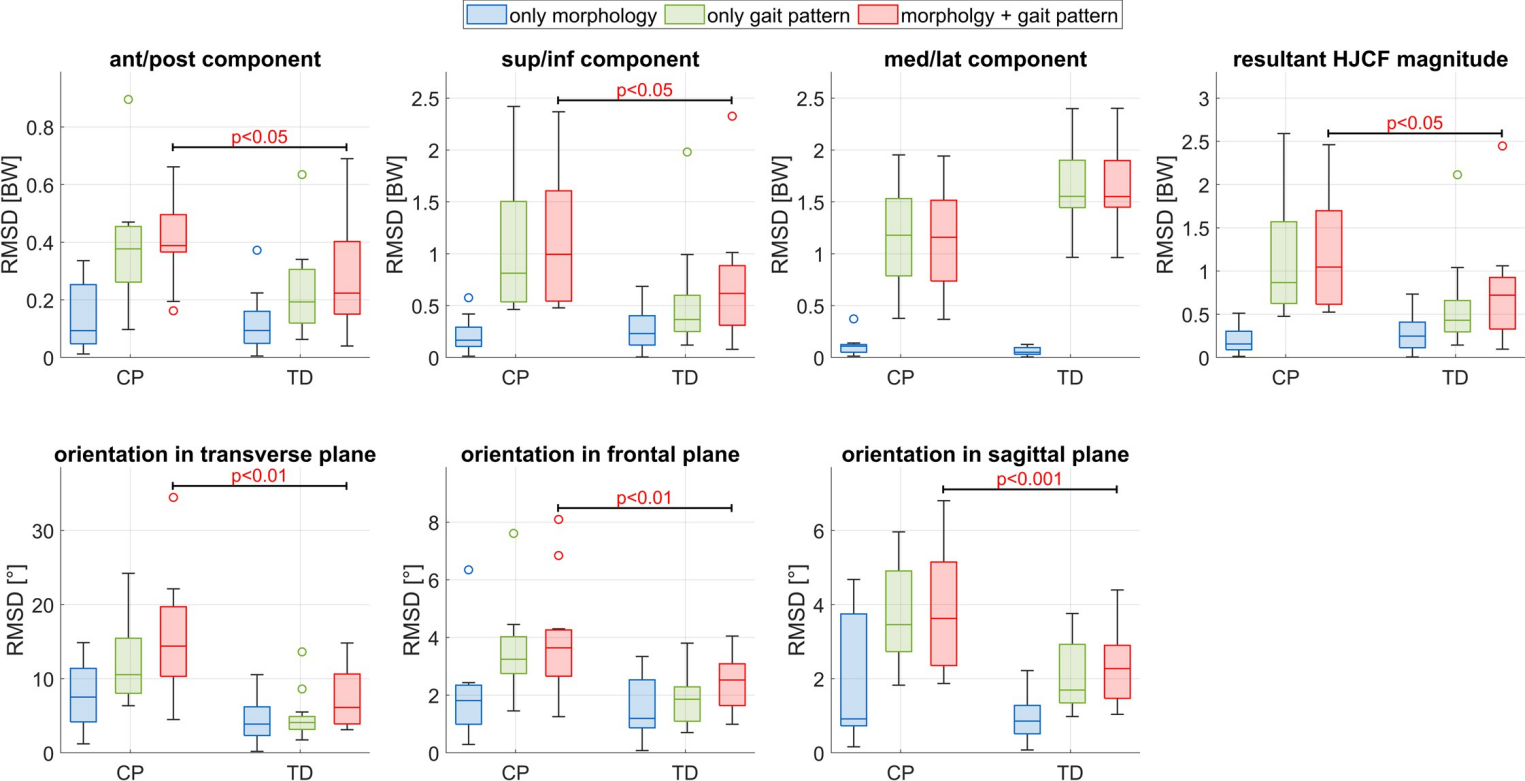
Contribution of the femoral morphology (blue), the gait pattern (green) and the combined contribution of morphology and gait pattern (red) on the asymmetry of HJCF magnitude and orientation during the stance phase.

Asymmetries in HJCF magnitude (anterior, inferior and resulting) and orientation (all planes) were significantly larger (p<0.05, effect size = 0.9±0.26, power = 0.69±0.17 for HJCF magnitude; p<0.05, effect size = 1.26±0.17, power = 0.92±0.06 for HJCF orientation) in CP compared to TD participants (Fig 6). In both groups (TD and CP), the gait pattern had a significantly larger (p<0.001, effect size = 2.48, power>0.99 for HJCF magnitude; p<0.05, effect size = 0.73, power>0.99 for HJCF orientation) impact on asymmetries in HJCF magnitudes and orientations compared to the morphology. For the HJCF orientation, a significant interaction effect (p<0.05) was observed showing that in the CP group the gait pattern has an even higher contribution to the HJCF asymmetry than the femoral morphology.

## Discussion

The purpose of this study was to comprehensively analyze HJCF asymmetries in children with CP and TD children and to evaluate if the subject-specific gait pattern or the femoral morphology is the main contributor to these asymmetries. In general, HJCF asymmetries were significantly higher in CP compared to TD children except for the medial/lateral component. In agreement with our hypothesis, the gait pattern had a larger influence on the HJCF asymmetry than the femoral morphology in both groups.

As anticipated, our findings showed that HJCF asymmetries are higher in CP compared to TD children. We expected this finding due to higher gait asymmetries in CP compared to TD children [11]. The standard deviations in HJCF asymmetries in the CP group were very high which indicates that some participants were similar to the TD group and others were far outside the TD range. This is in agreement with previous work [18], which showed that some children with CP have typical HJCF, whereas others have HJCF outside the TD range. Future work based on a larger cohort of CP participants is needed to identify how different pathological gait pattern, e.g. in-toing gait or crouch gait, or CP diagnosis, e.g. hemiplegic or diplegic CP, affect HJCF asymmetries.

The overall femoral asymmetry, i.e. MAS, was as expected higher in children with CP compared to TD children. Furthermore, the NSAs were significantly higher in CP compared to TD participants. However, against our expectation based on previous studies [2, 45], the AVAs were significantly lower in our CP compared to our TD participants. Typically, the AVA decreases with age in TD children and remains higher in children with CP [2, 45]. There was no significant difference in age between our CP and TD groups, which could explain the observed difference. The AVAs of our TD participants were within the range of TD values reported in a study based on 508 participants [46]. Hence, we assume that the low number of participants in our study is responsible for the observed higher AVAs in our TD compared to our CP participants.

In children with CP single event multi-level surgeries including de-rotation osteotomies and muscle-tendon-lengthening surgeries are often used to correct femoral deformities, improve the child’s gait pattern and prevent the development of further deformities [23–25]. The gained insights from our study suggest that normalizing the gait pattern should be a high priority of clinical interventions and might be even more important than correcting the bony deformities. However, de-rotation osteotomies normalize the lever arms of muscles [23, 24], which might be necessary to enable a typical walking pattern. Hence, clinical interventions should target static, anatomical impairments, i.e. bony deformities, and dynamic, functional impairments, i.e. pathological gait pattern. Based on our findings, solely the correction of bony deformities is unlikely to stop the development of further deformities. Therefore, an intervention should only be judged successful if the gait pattern improves additionally to the anatomical correction. Yadav et al. (2021) performed predictive simulations based on a mechanobiological model and concluded that the posterior and the lateral HJCF components highly affect the change of the NSA and AVA. Hence, a normalization of these HJCF components seems to be of high importance. Further studies are needed to identify which gait modifications and clinical interventions could achieve the desired normalization of the HJCF.

Our study included the following limitations. First, only the femoral morphology was personalized in our musculoskeletal models. Tibial morphology could not be personalized because MRI images of the tibia were not available for all participants. Changes of tibial geometry alters the moment arms of the shank muscles and consecutively has an impact on the estimation of muscle and knee joint contact forces. Due to the bottom-up approach used to estimate joint contact forces in OpenSim [37], changes in the knee joint contact force would also affect HJCF. However, tibial torsion only affects lever arms of a limit number of muscles and therefore we assume the impact on HJCF is negligible. Further studies which investigate the impact of tibial torsion on HJCF should be carried out to verify our assumption. Second, the HJCF could not be measured in-vivo and therefore were estimated with musculoskeletal simulation. The shape and magnitude of our HJCF were, however, in agreement with previous studies [16, 18, 47–49]. Third, although we showed that the gait pattern has a larger influence on HJCF than the femoral morphology, it has to be mentioned that the femoral geometry itself influences the moment arms of several hip muscles [9, 50, 51] and therefore might be the reason why some children are not able to walk with a typical walking pattern [10]. Fourth, mean kinematic and kinetic waveforms were calculated based on a different number of trials between participants due to a low number of valid force plate strikes in some children with CP. Although this is standard practice in clinical and research settings [52], it potentially had a small impact on the obtained mean waveforms. Fifth, our intention was not to find the cause of femoral deformities but to identify the main contributor to asymmetric HJCFs. Pathological gait patterns alter the HJCF which is the main biomarker that determines femoral bone growth [14–16, 18]. Future studies including predictive musculoskeletal and mechanobiological simulations [18, 53] are needed to comprehensively evaluate the impact of femoral morphology on the walking ability and investigate the reasons for femoral deformities.

To conclude, this study comprehensively analyzed HJCF asymmetries in CP and TD children and identified the contribution of subject-specific femoral morphology and gait pattern to the asymmetry of hip loading. The asymmetric gait pattern had a larger influence on HJCF asymmetries than the asymmetric femoral morphology. Gait asymmetries and therefore also HJCF asymmetries were larger in CP compared to TD participants. Bone is adaptive to mechanical loading [12, 54–56] and pathological HJCF will likely lead to pathological femoral growth [18]. Hence, in patients with femoral deformities it is of utmost importance to correct the functional impairment, i.e. patient-specific gait pattern, with the aim to normalize loading and femoral growth. Whether normalization of the gait pattern can be achieved solely with physical therapy (e.g. gait retraining) or in combination with surgical interventions depends on the individual patient and was beyond the scope of our study. However, our findings highlight that pre- and post-intervention gait analysis should be performed to evaluate the success of an intervention.

All musculoskeletal models and the simulation results are published on https://simtk.org/projects/bone_gait_load to allow peers to further investigate the data.

## Supporting information

S1 File This file contains CP participants’ details, additional plots on the correlation between asymmetry of femoral morphology and asymmetry of HJCF and each CP participants’ kinematic. (DOCX)
