# Supplementary material for: The gait pattern and not the femoral morphology is the main contributor to asymmetric hip joint loading
Source: PLoS One. 2023 Sep 26;18(9):e0291789. doi: 10.1371/journal.pone.0291789 (PMC10522038; doi:10.1371/journal.pone.0291789)
Supplement: S1 File — (DOCX) [file pone.0291789.s001.docx]

Supplementary material of

The gait pattern and not the femoral morphology is the main contributor to asymmetric hip joint loading

Willi Koller, Arnold Baca, Hans Kainz

Inhalt

[1. Participant details 1](#_Toc136505725)

[2. Gait kinematics of participants with CP 1](#_Toc136505726)

[3. Correlation between asymmetry of femoral morphology and asymmetry of HJCF 8](#_Toc136505727)

# Participant details

Table S1: Shows the diagnosis and walking style pattern of the participants with CP. The walking style was determined based on visual inspection of the particpants kinematics and classified by types of CP gait patterns described in Sloot, L. H. (2016). Advanced technologies to assess motor dysfunction in children with cerebral palsy. [PhD-Thesis - Research and graduation internal, Vrije Universiteit Amsterdam]. <https://research.vu.nl/ws/portalfiles/portal/42157340/>

|  | **Diagnosis from clinicians** | **GMFCS** | **walking style** | |
| --- | --- | --- | --- | --- |
|  |  |  | **left** | **right** |
| CP01 | left hemiplegic | 1 | crouch gait | normal |
| CP02 | right hemiplegic | 1 | normal | equinus jump gait |
| CP03 | right hemiplegic | 1 | normal | true equinus |
| CP04 | spastic diplegia | 2 | crouch gait | crouch gait |
| CP05 | spastic diplegia | 2 | apparent equinus | apparent equinus |
| CP06 | spastic diplegia | 2 | equinus jump gait | equinus jump gait |
| CP07 | diplegia | 2 | apparent equinus | apparent equinus |
| CP08 | diplegia | 2 | crouch gait | crouch gait |
| CP09 | right hemiplegic | 2 | normal | true equinus |
| CP10 | spastic diplegia | 2 | equinus jump gait | equinus jump gait |
| CP11 | spastic diplegia | 2 | equinus jump gait | equinus jump gait |
| CP12 | right hemiplegic | 2 | normal | true equinus |

# Gait kinematics of participants with CP

Gait patterns varies widely in the cohort of children with CP. For completeness and to allow a more detailed discussion on the results of this paper plots showing the gait kinematics of each participant with CP are included.


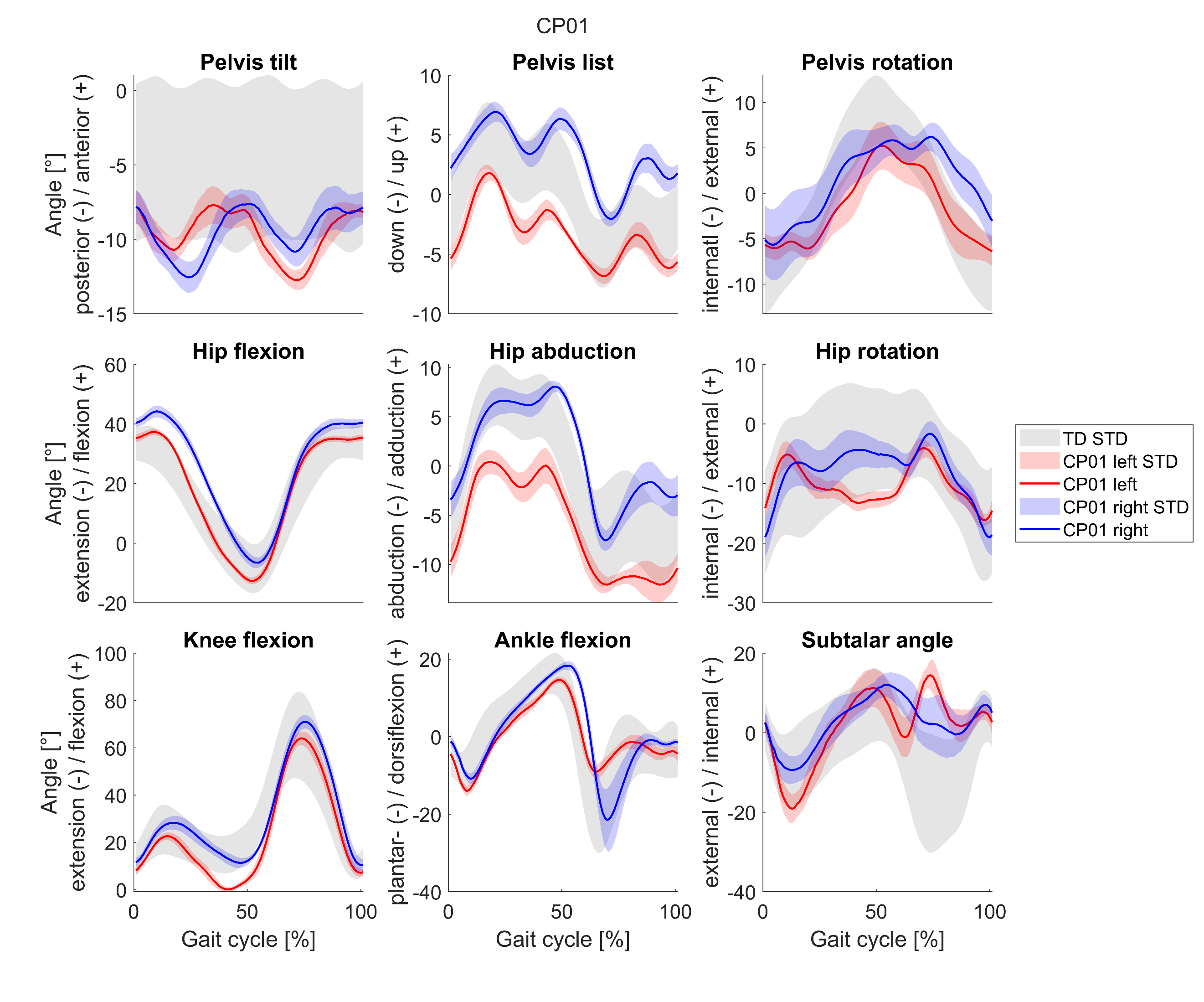

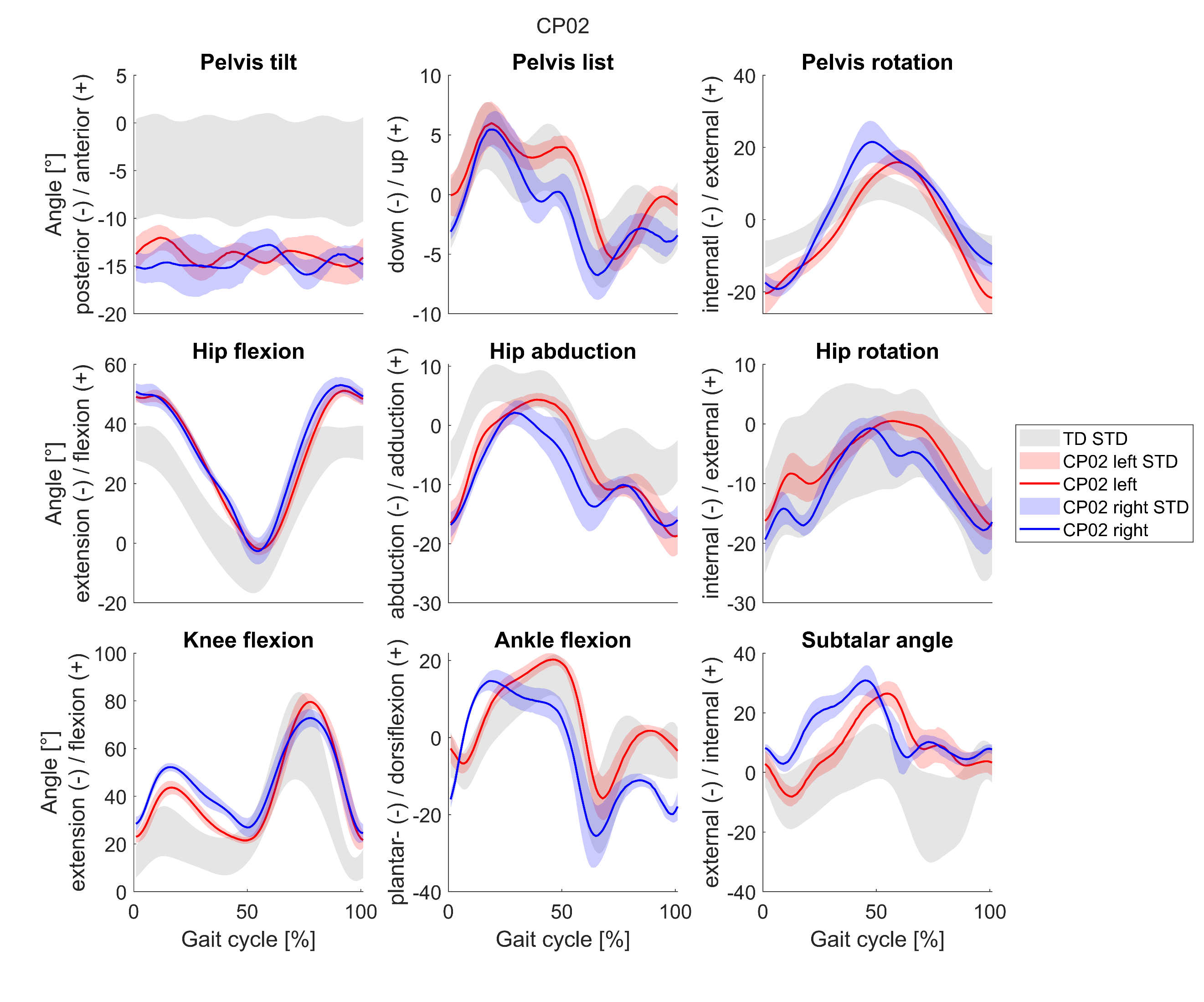

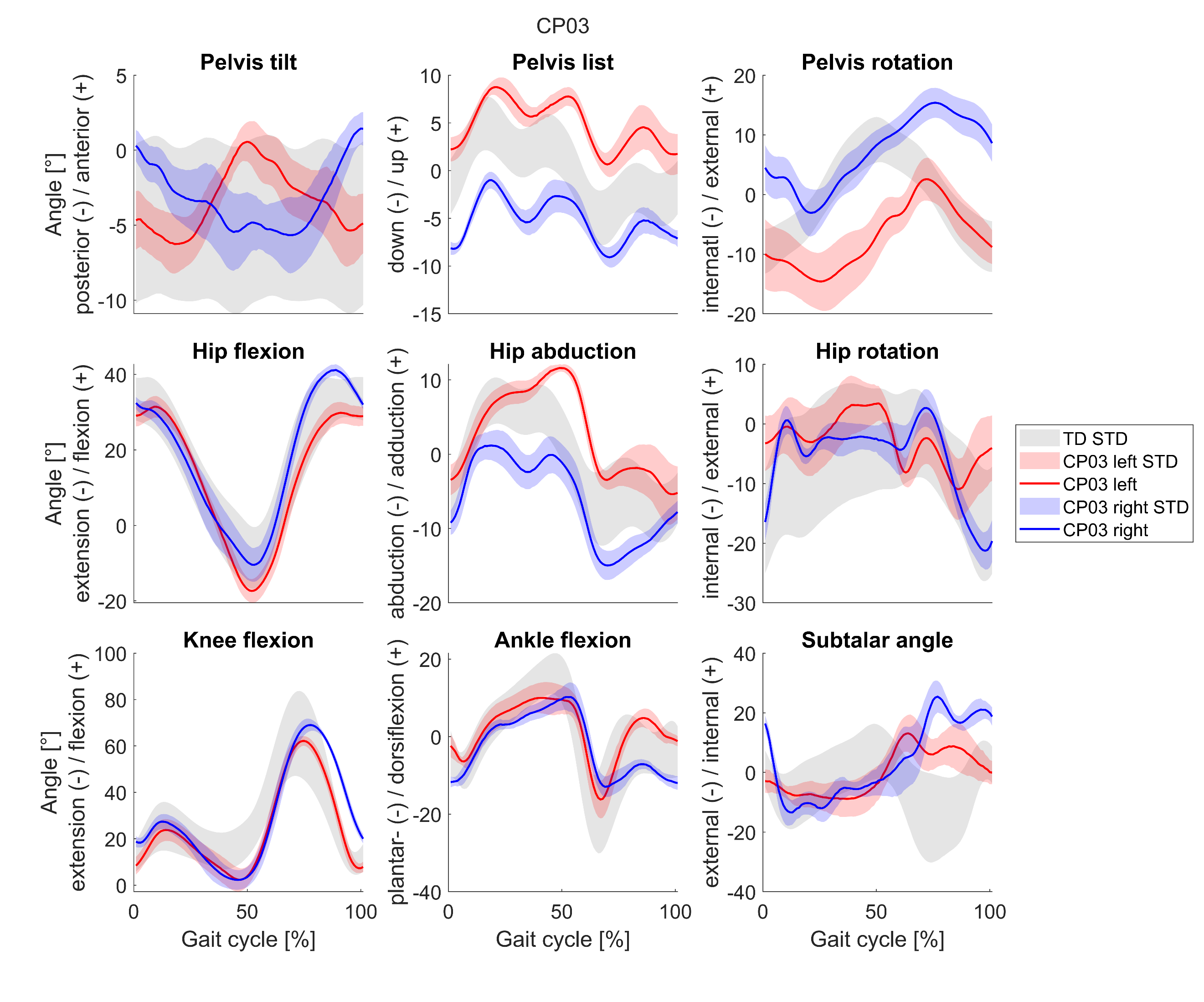

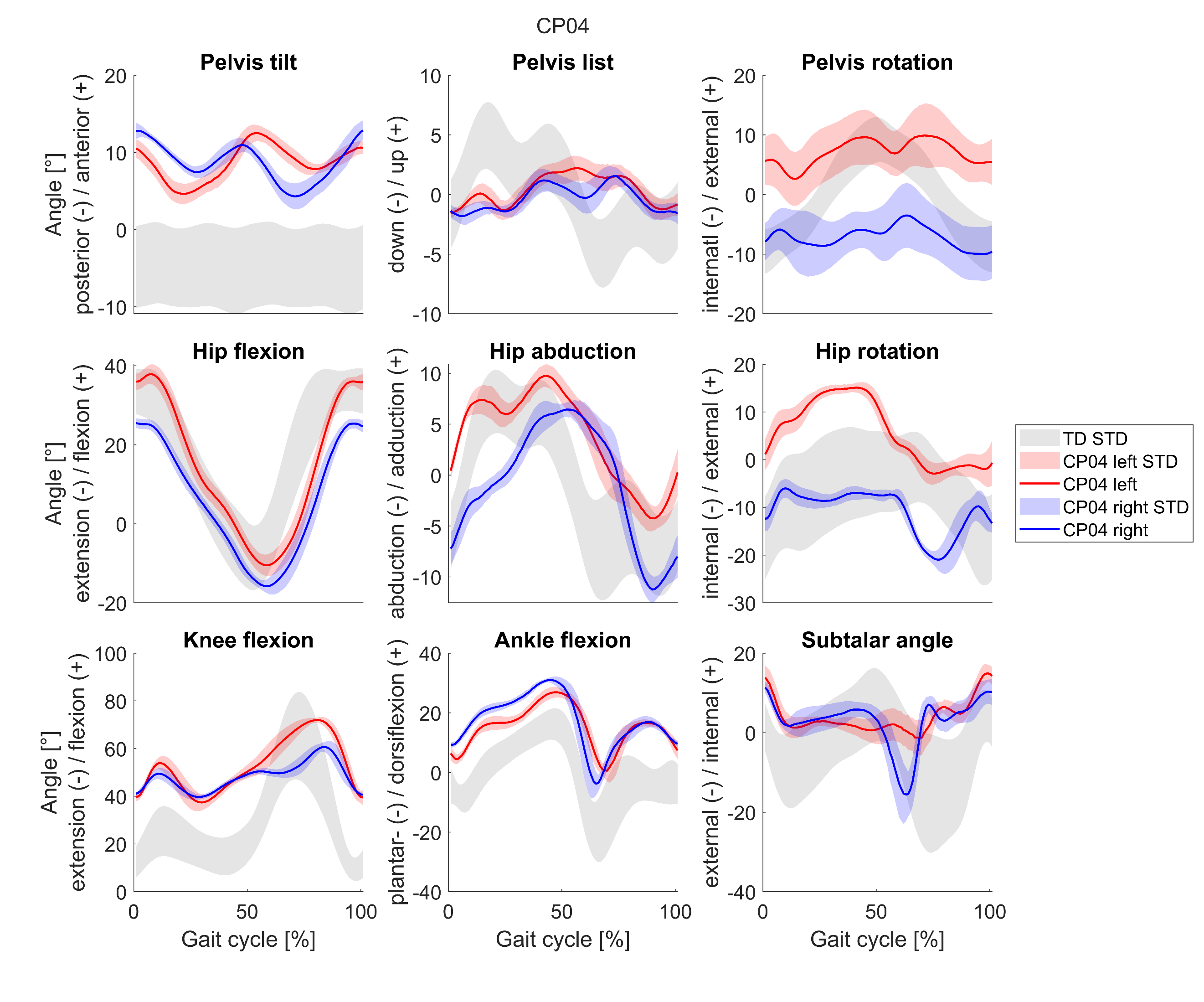


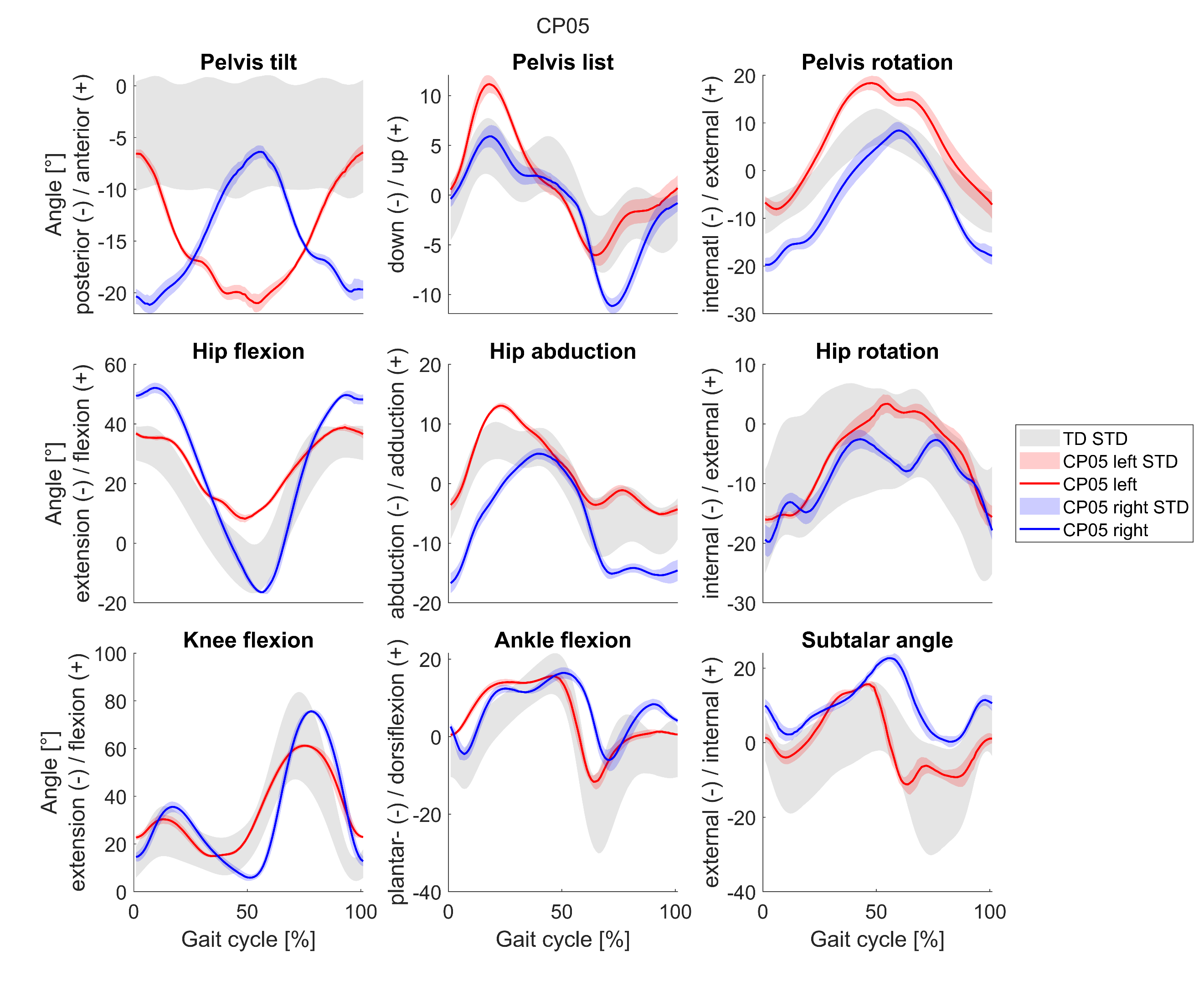

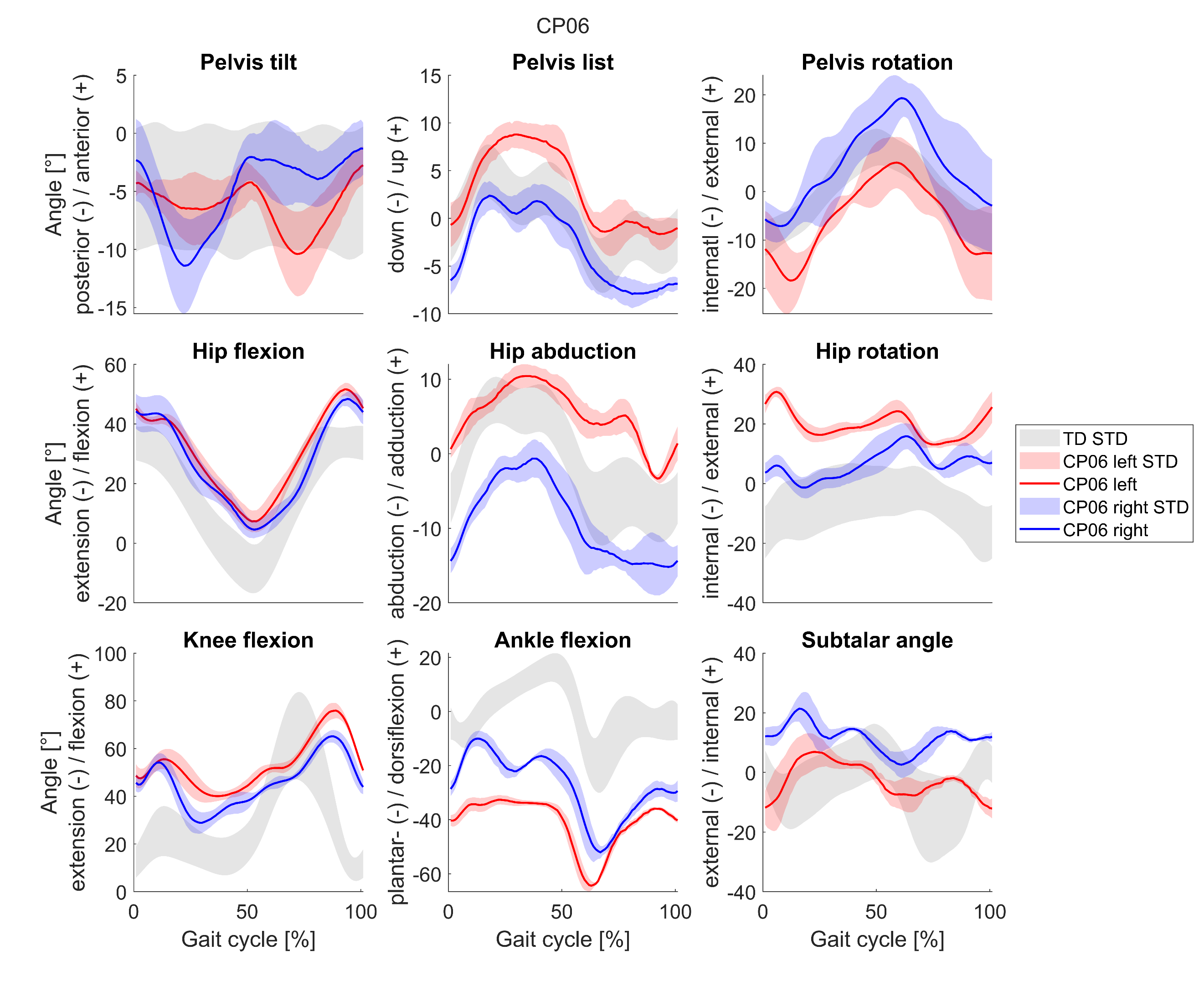

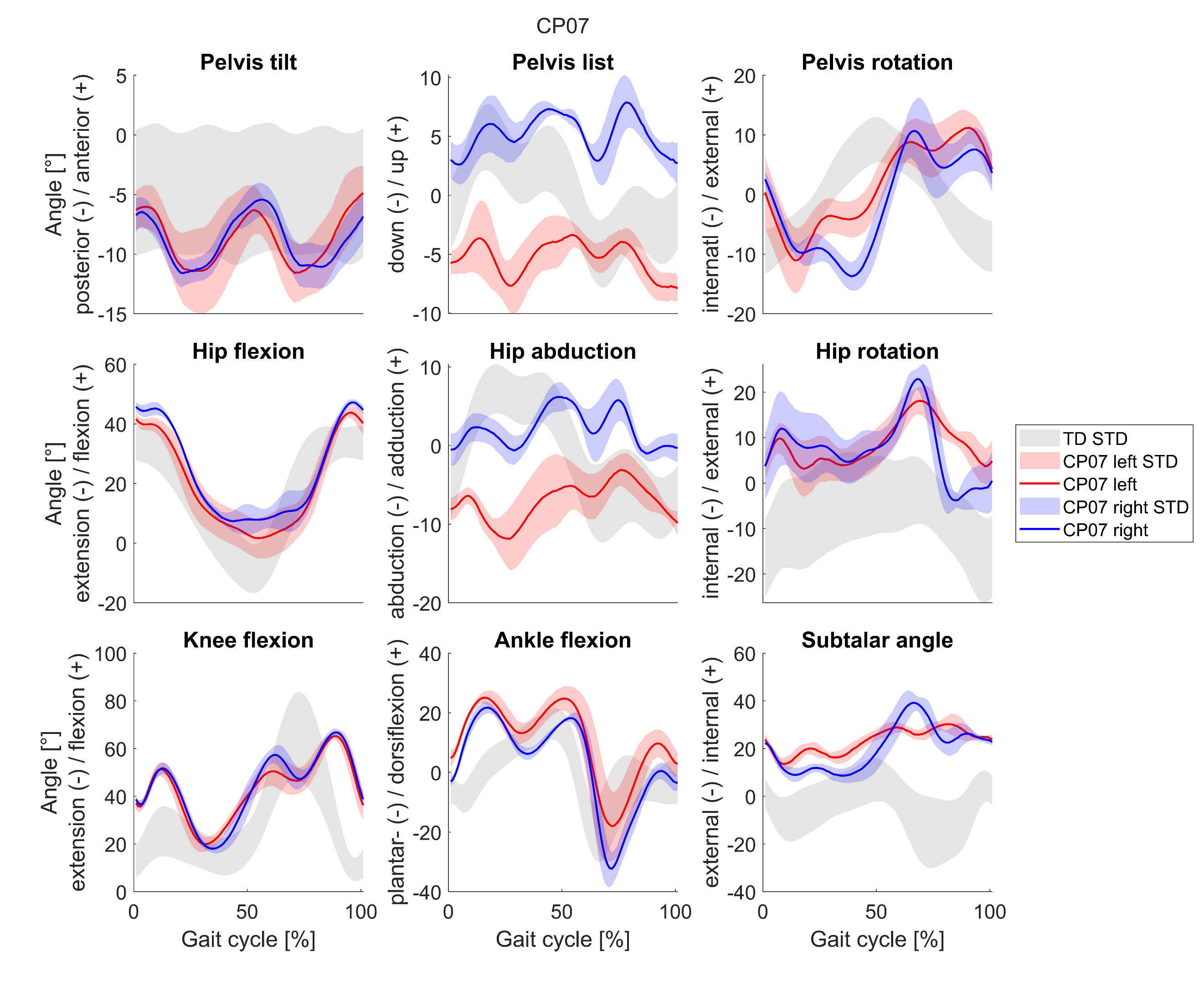

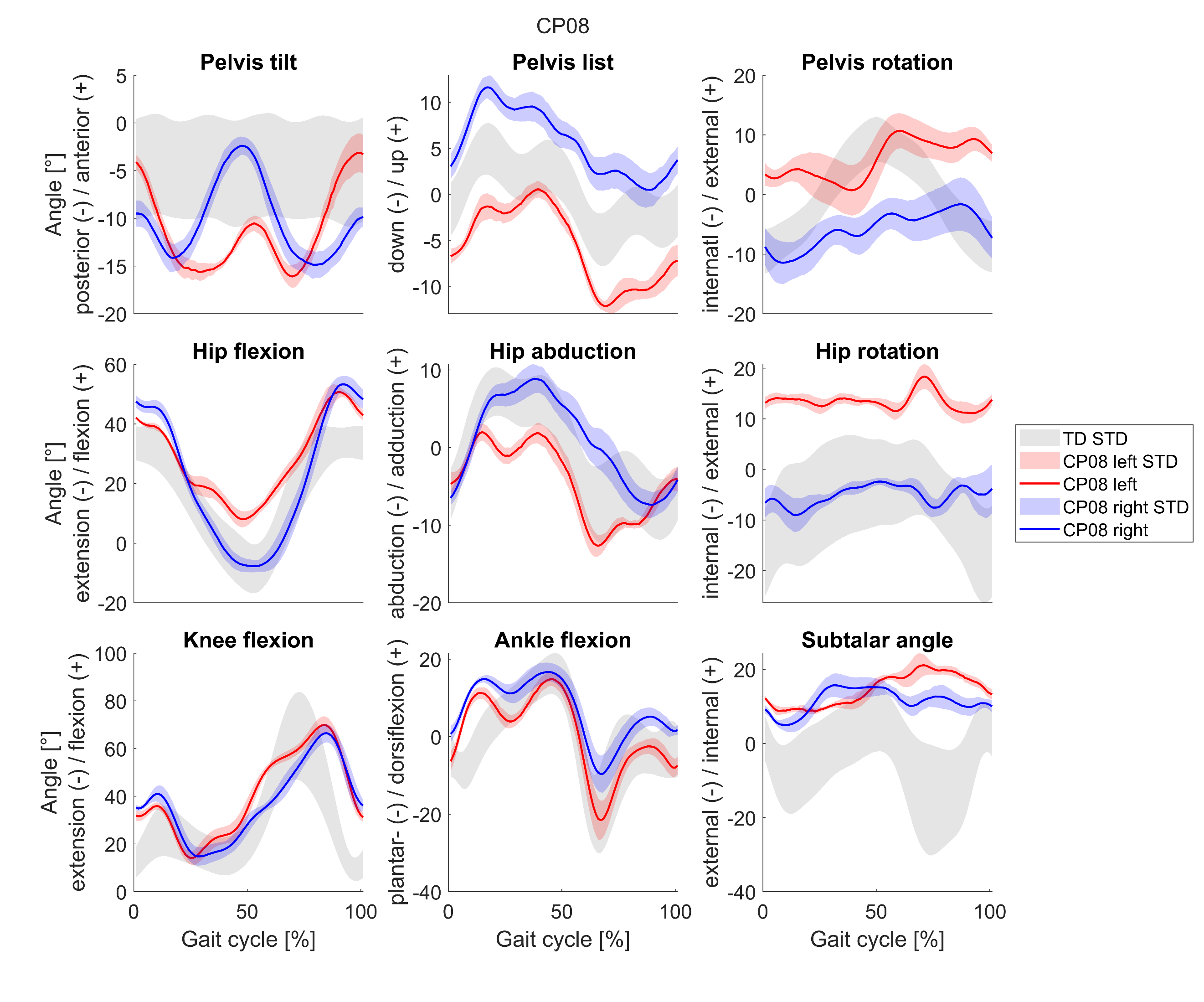

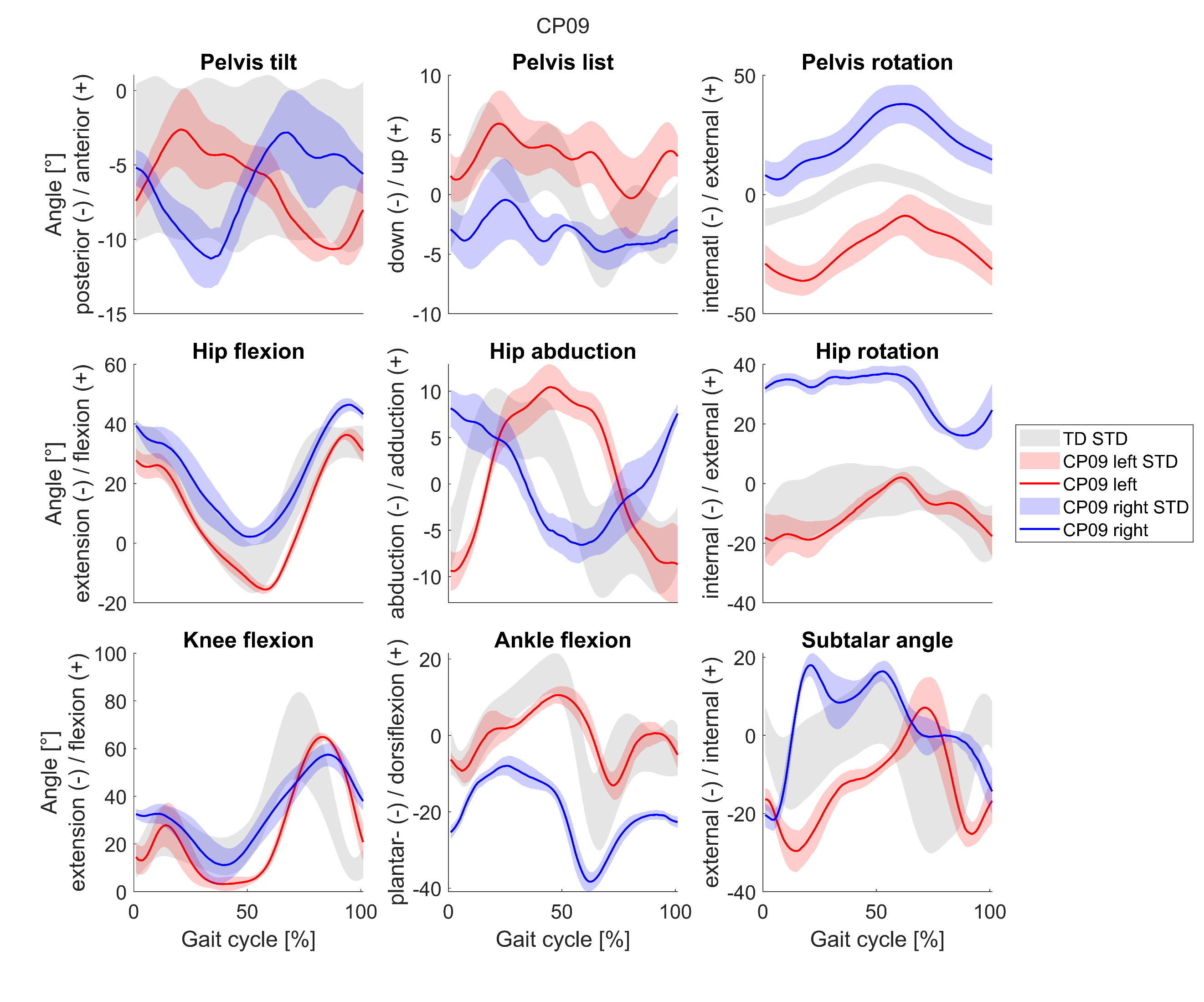

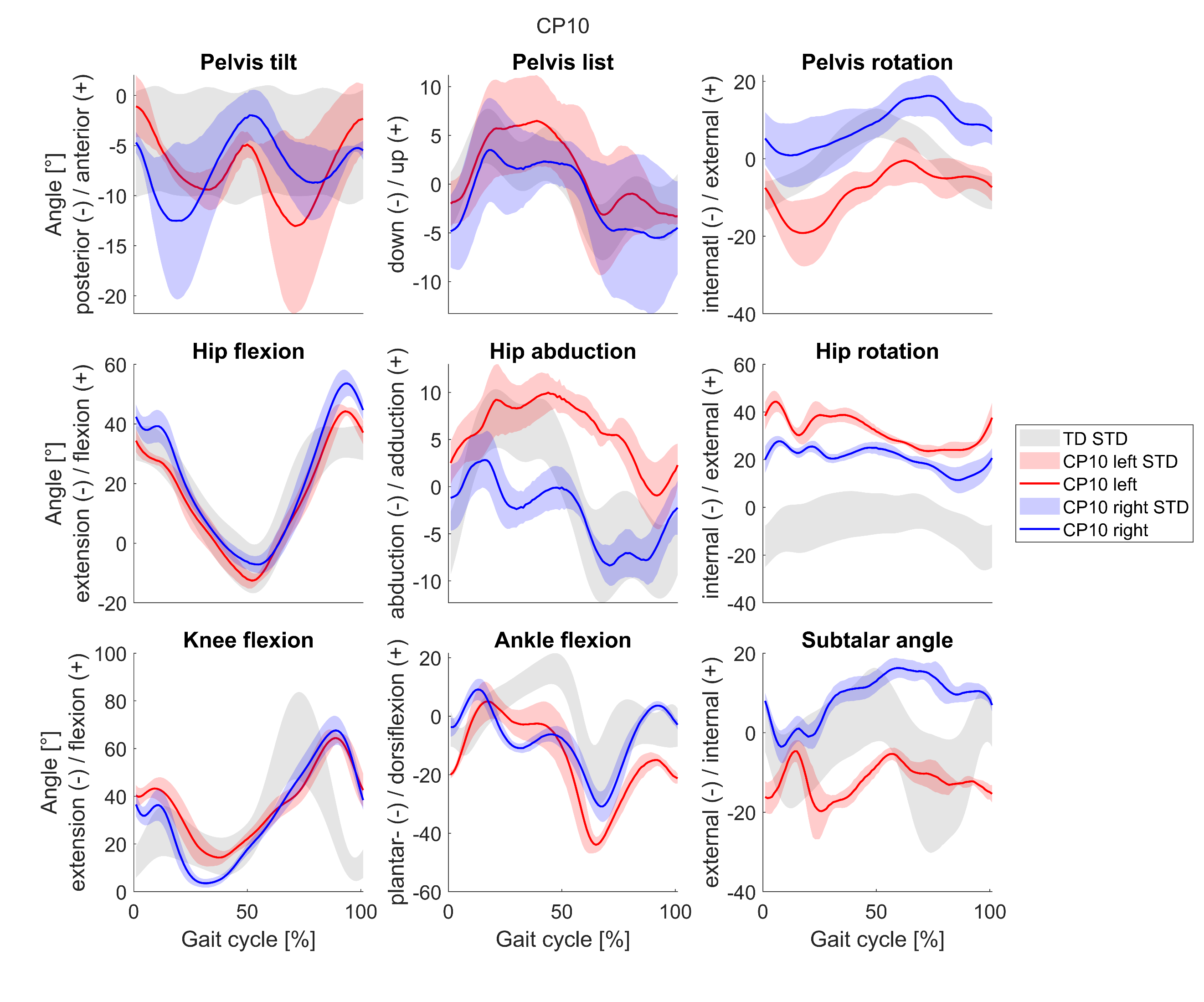

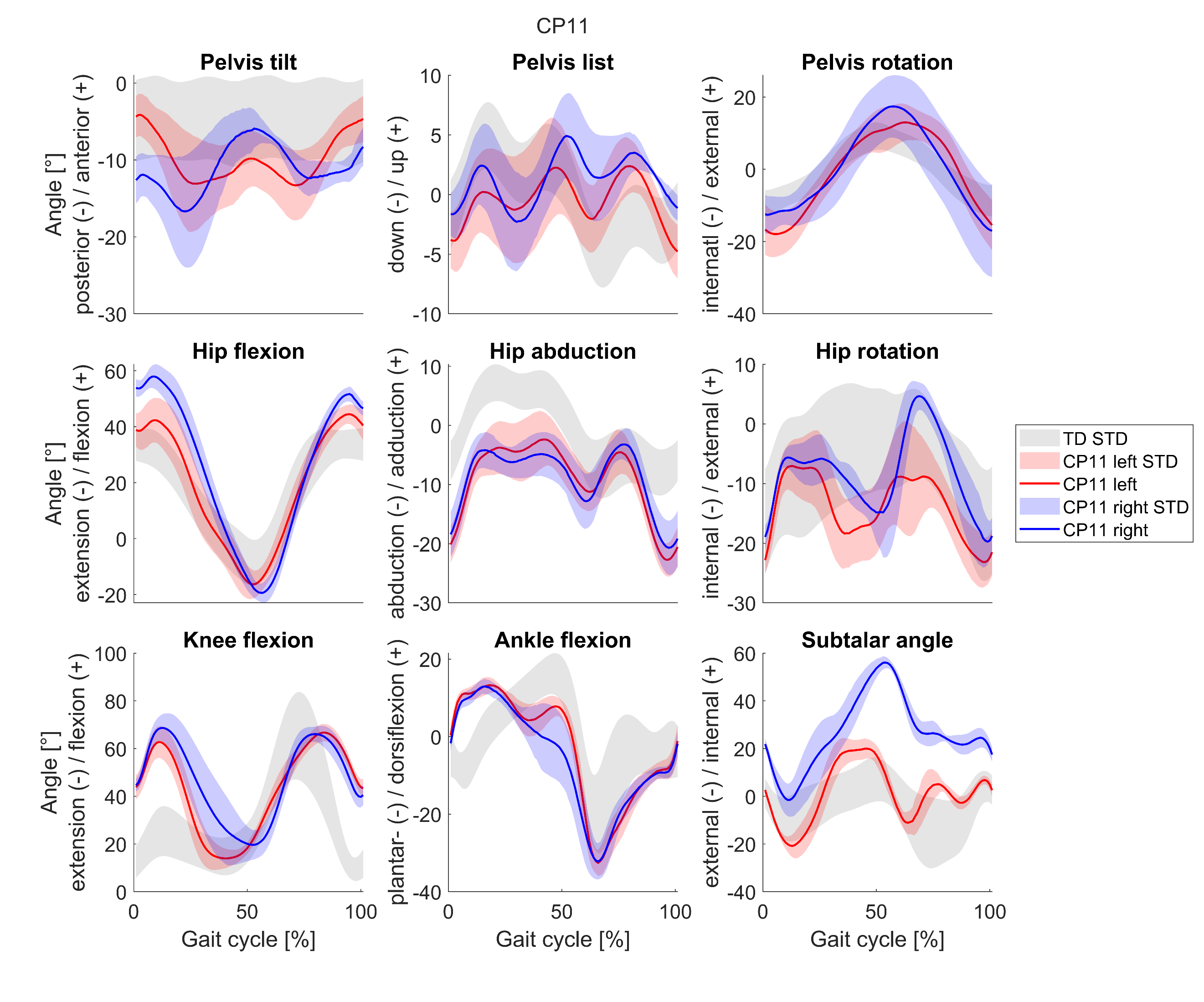

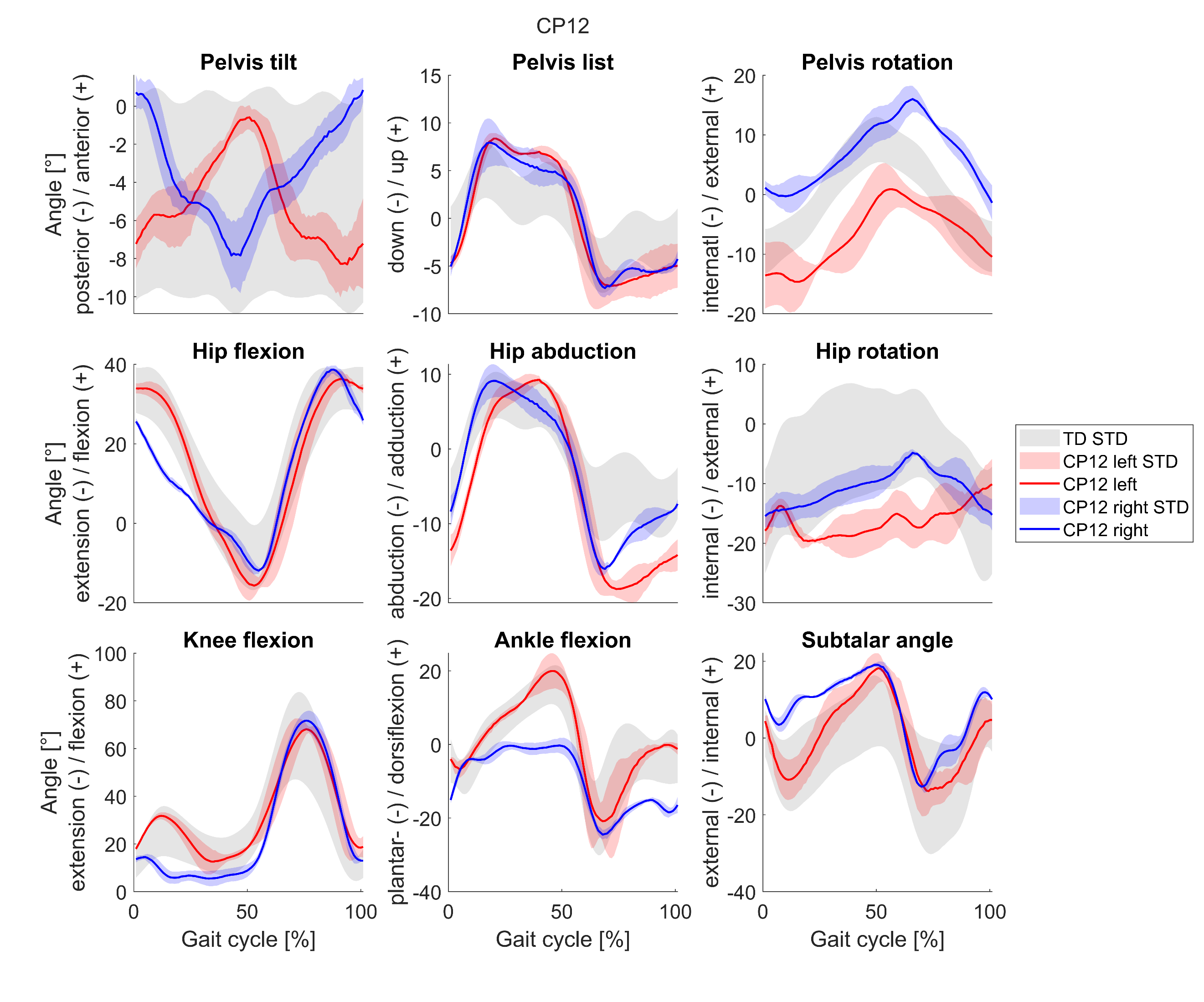


# Correlation between asymmetry of femoral morphology and asymmetry of HJCF

Methods: We evaluated if there are significant linear regression correlations between the asymmetry in femoral morphology (ΔNSA, ΔAVA and MAS) and the asymmetry of HJCF magnitude and/or orientation.

Results: Mainly, linear correlations were found between the asymmetries of HJCF due to the participants’ femoral morphology and the MAS, ΔNSA and ΔAVA (Table S1). The asymmetries in AVA (ΔAVA) account for asymmetries in posterior, inferior and resultant HJCF whereas asymmetries in NSA (ΔNSA) were the main contributor for HJCF asymmetries in the lateral direction (Figures S1 – S7).

Discussion: With these linear correlations we evaluated if asymmetries in HJCF magnitude and orientation can be predicted from the asymmetry of the femoral geometry. The high coefficients of determination (R2) between the morphology and the asymmetries of HJCF due to morphology serve as a validation test for our simulation approach. In simpler terms, we examined if there is a notable relationship between the morphology and the asymmetry of the HJCF due to the morphology. For the combined (i.e. “real”) HJCF we found no high correlations which is in agreement with the other tests which indicate that the gait pattern is much more important that the femoral geometry (NSA, AVA, MAS).

Table S2. R^2^ of significant linear correlations between femoral morphology and asymmetry of HJCF magnitude and orientation.

| **R^2^ if p<0.05** | | | **HJCF magnitude asymmetry** | | | | **HJCF orientation asymmetry** | | |
| --- | --- | --- | --- | --- | --- | --- | --- | --- | --- |
|  |  |  | **posterior** | **inferior** | **lateral** | **resultant** | **transverse** | **frontal** | **sagittal** |
| **due to morphology** | MAS | CP | 0.74 |  |  |  | 0.91 |  | 0.94 |
|  |  | TD | 0.80 | 0.61 | 0.53 | 0.64 | 0.90 | 0.73 | 0.56 |
|  | ΔNSA | CP |  |  | 0.48 |  | 0.60 | 0.68 | 0.52 |
|  |  | TD |  |  | 0.70 |  |  | 0.44 |  |
|  | ΔAVA | CP | 0.78 |  |  |  | 0.80 |  | 0.89 |
|  |  | TD | 0.79 | 0.51 |  | 0.57 | 0.82 | 0.48 | 0.67 |
| **due to gait pattern** | MAS | CP |  |  |  |  |  |  |  |
|  |  | TD |  |  |  |  |  |  |  |
|  | ΔNSA | CP |  |  |  |  |  |  |  |
|  |  | TD |  |  |  |  |  |  |  |
|  | ΔAVA | CP |  |  |  |  |  |  |  |
|  |  | TD |  |  |  |  |  |  |  |
| **combined** | MAS | CP |  |  |  |  |  | 0.43 |  |
|  |  | TD |  |  |  |  | 0.42 |  | 0.46 |
|  | ΔNSA | CP |  |  |  |  |  |  |  |
|  |  | TD |  |  |  |  |  |  |  |
|  | ΔAVA | CP |  |  |  |  | 0.49 | 0.39 |  |
|  |  | TD |  |  |  |  | 0.34 |  | 0.42 |


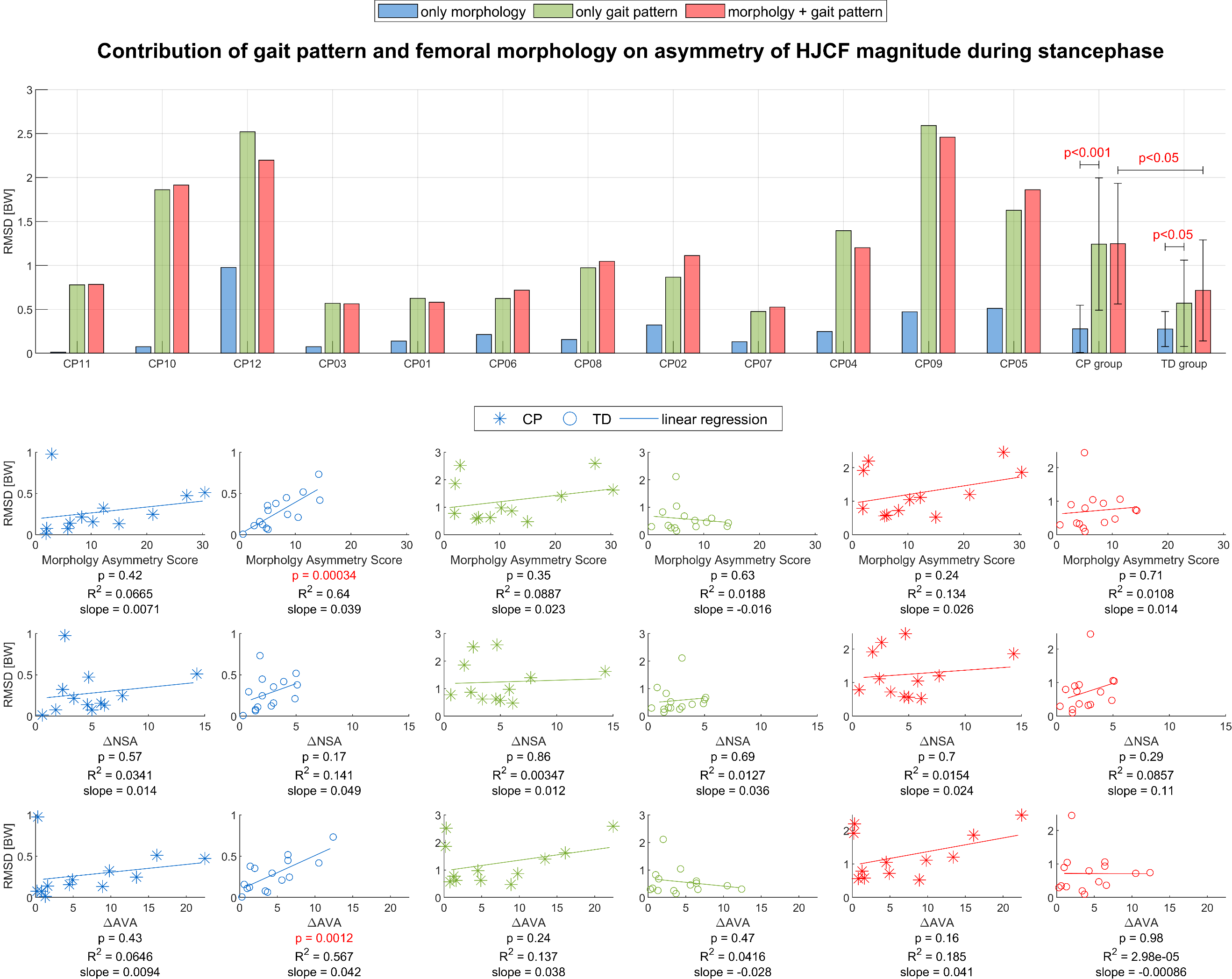


S1 Fig. RMSD between left and right resultant HJCF due to the femoral morphology (blue), the child’s gait pattern (green) and both combined (red). Below scatter plots showing the correlation between asymmetric HJCF and femoral morphology (MAS, ΔNSA, ΔAVA).


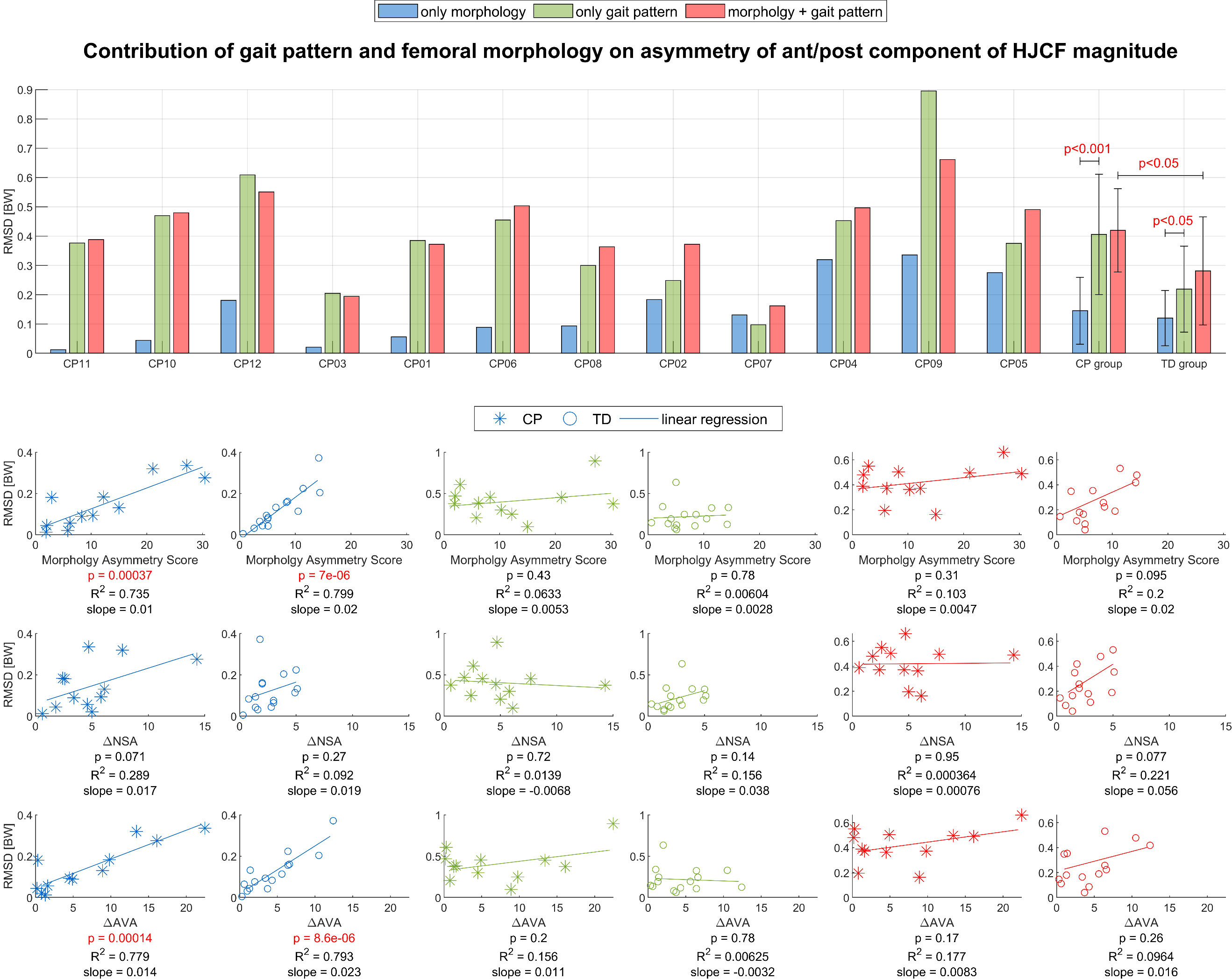


S2 Fig. RMSD between left and right anterior/posterior component of HJCF due to the femoral morphology (blue), the child’s gait pattern (green) and both combined (red). Below scatter plots showing the correlation between asymmetric HJCF and femoral morphology (MAS, ΔNSA, ΔAVA).


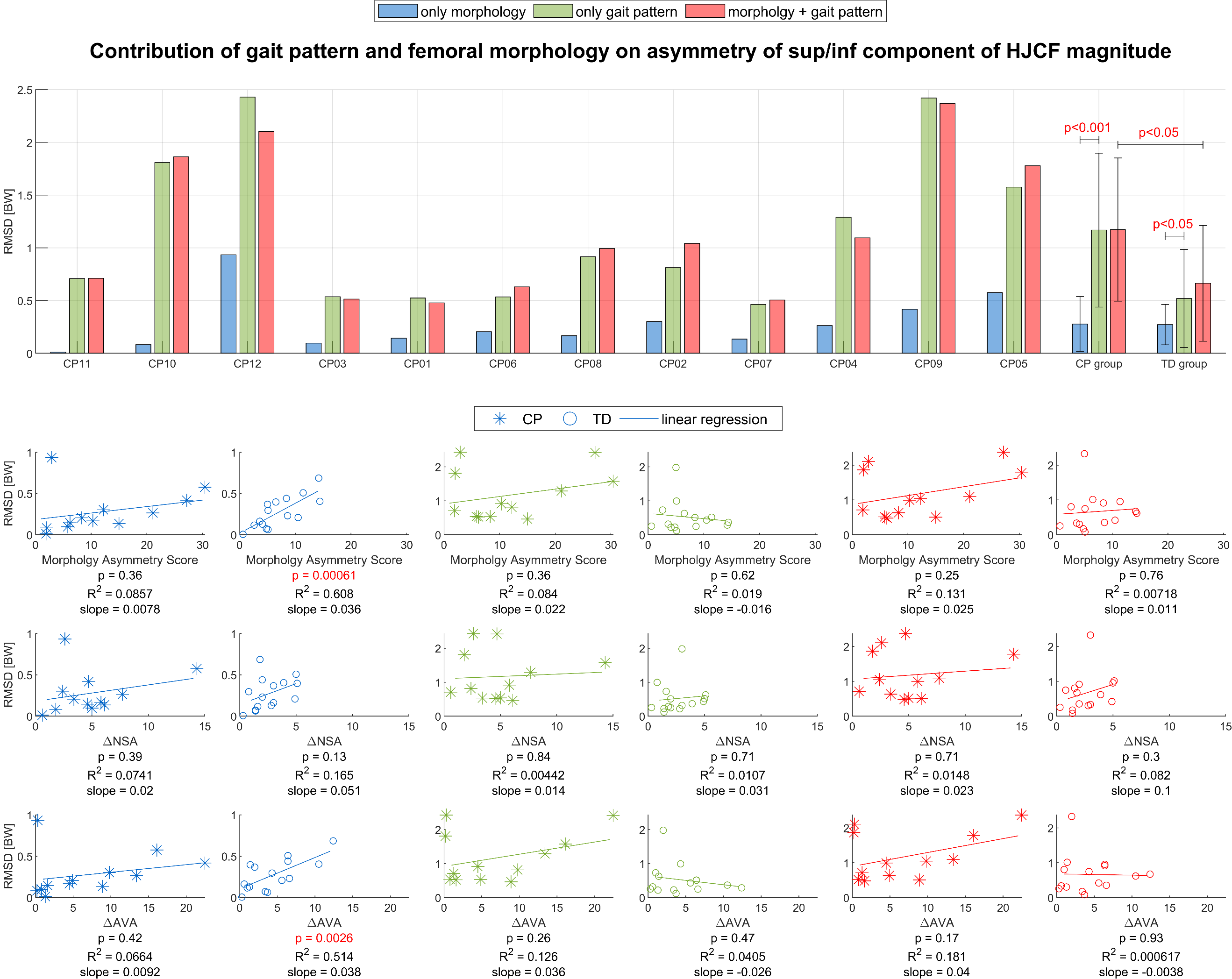


S3 Fig. RMSD between left and right superior/inferior component of HJCF due to the femoral morphology (blue), the child’s gait pattern (green) and both combined (red). Below scatter plots showing the correlation between asymmetric HJCF and femoral morphology


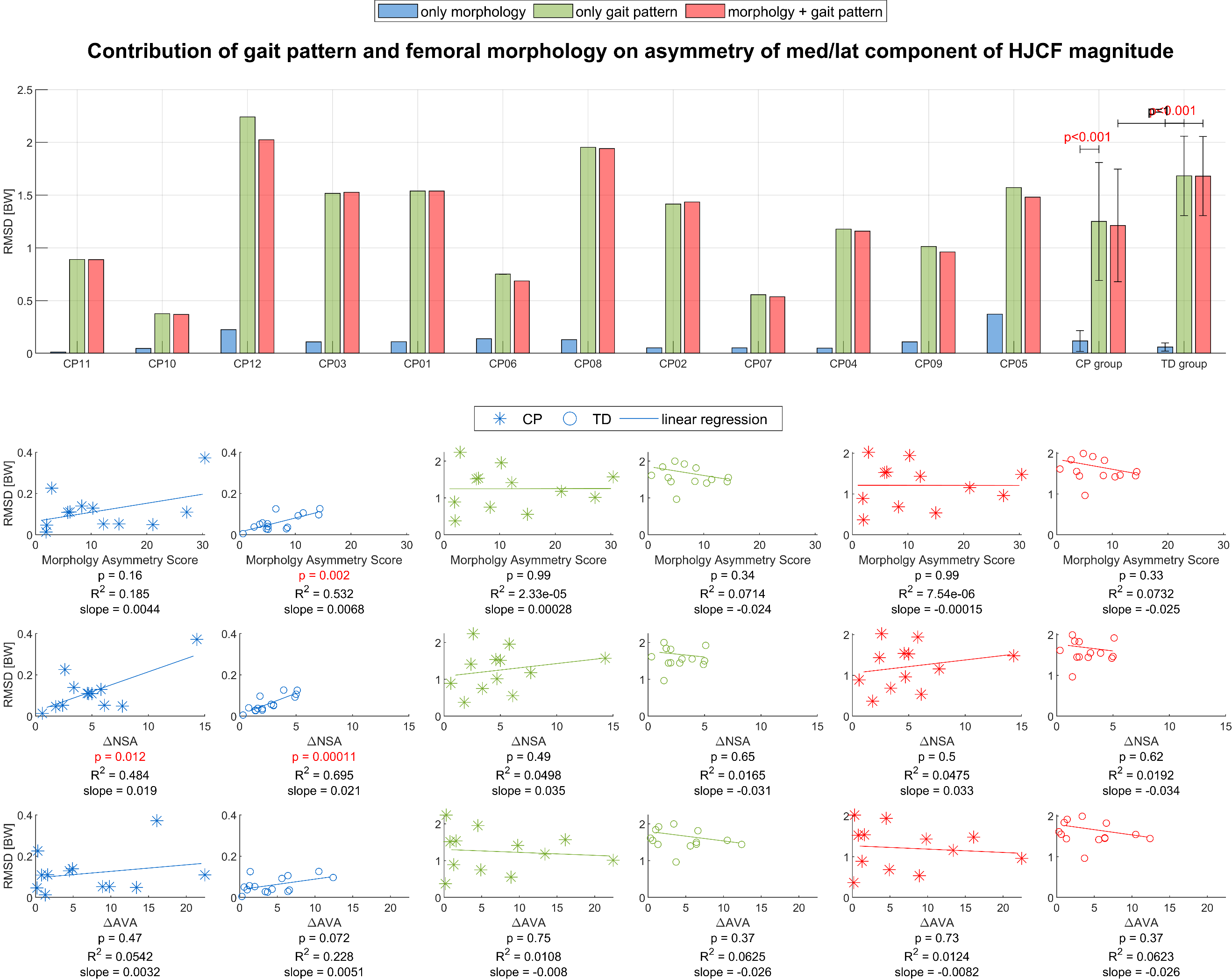


S4 Fig. RMSD between left and right medial/lateral component of HJCF due to the femoral morphology (blue), the child’s gait pattern (green) and both combined (red). Below scatter plots showing the correlation between asymmetric HJCF and femoral morphology


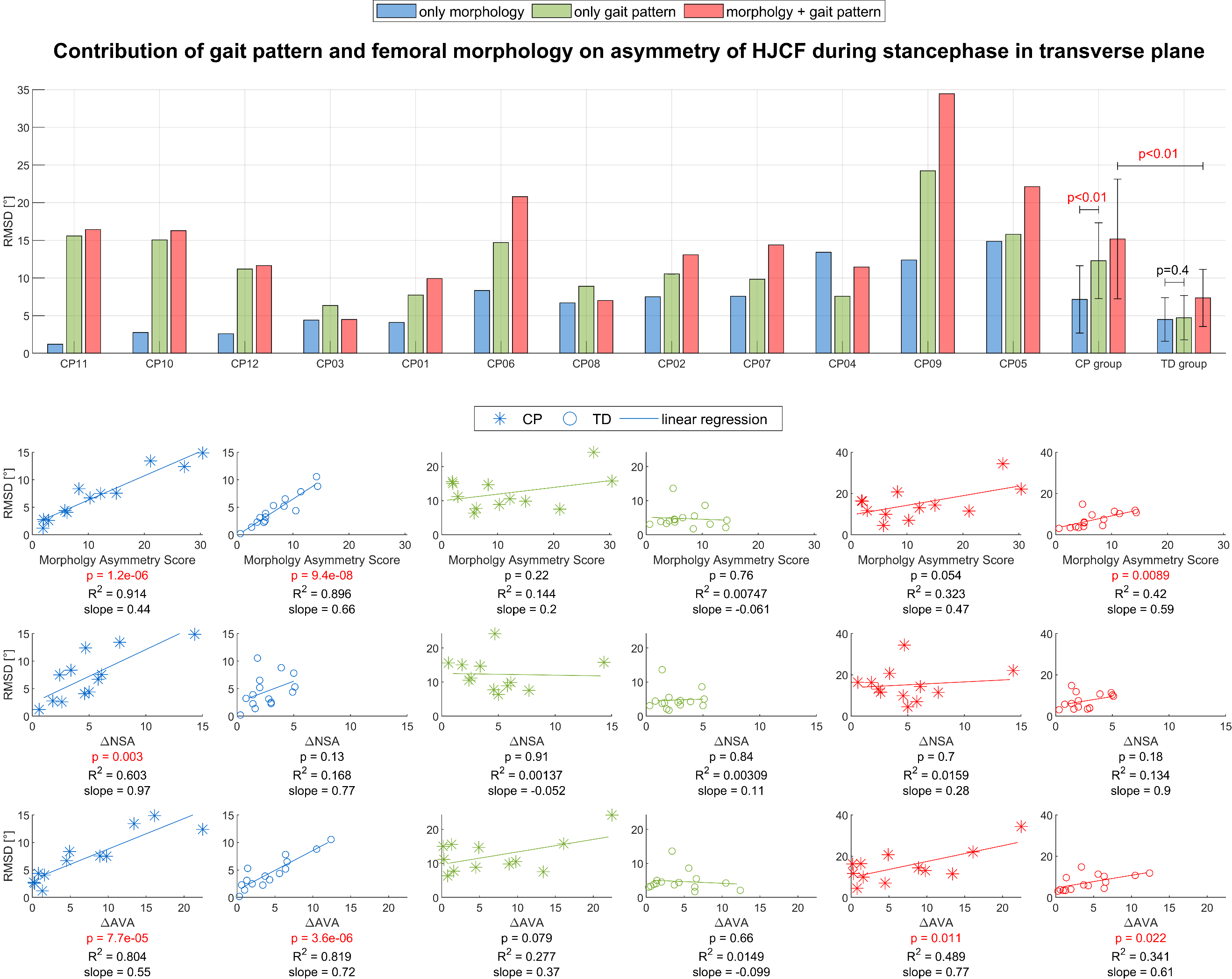


S5 Fig. RMSD between left and right HJCF orientation in transverse plane due to the femoral morphology (blue), the child’s gait pattern (green) and both combined (red). Below scatter plots showing the correlation between asymmetric HJCF and femoral morphology


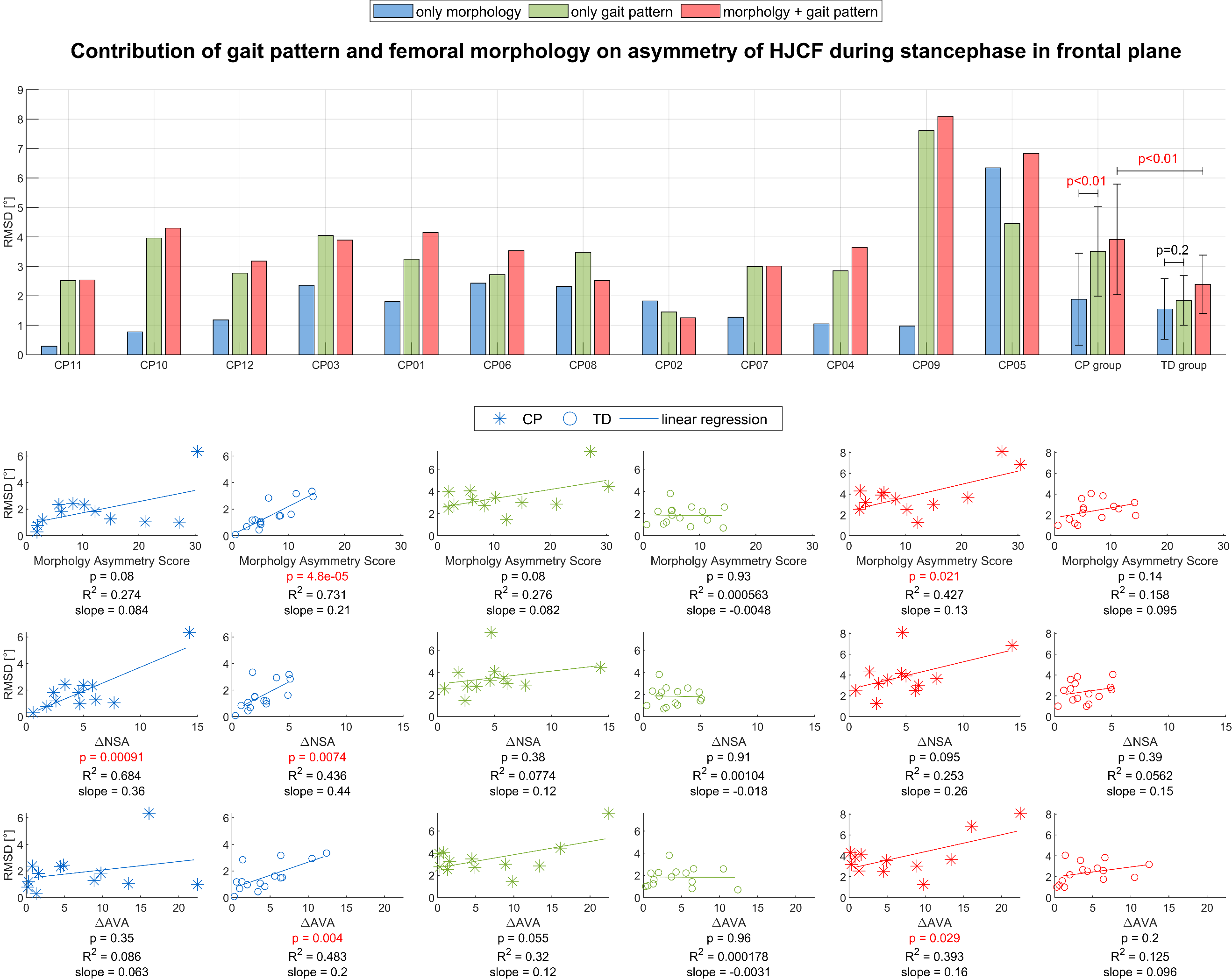


S6 Fig. RMSD between left and right HJCF orientation in frontal plane due to the femoral morphology (blue), the child’s gait pattern (green) and both combined (red). Below scatter plots showing the correlation between asymmetric HJCF and femoral morphology


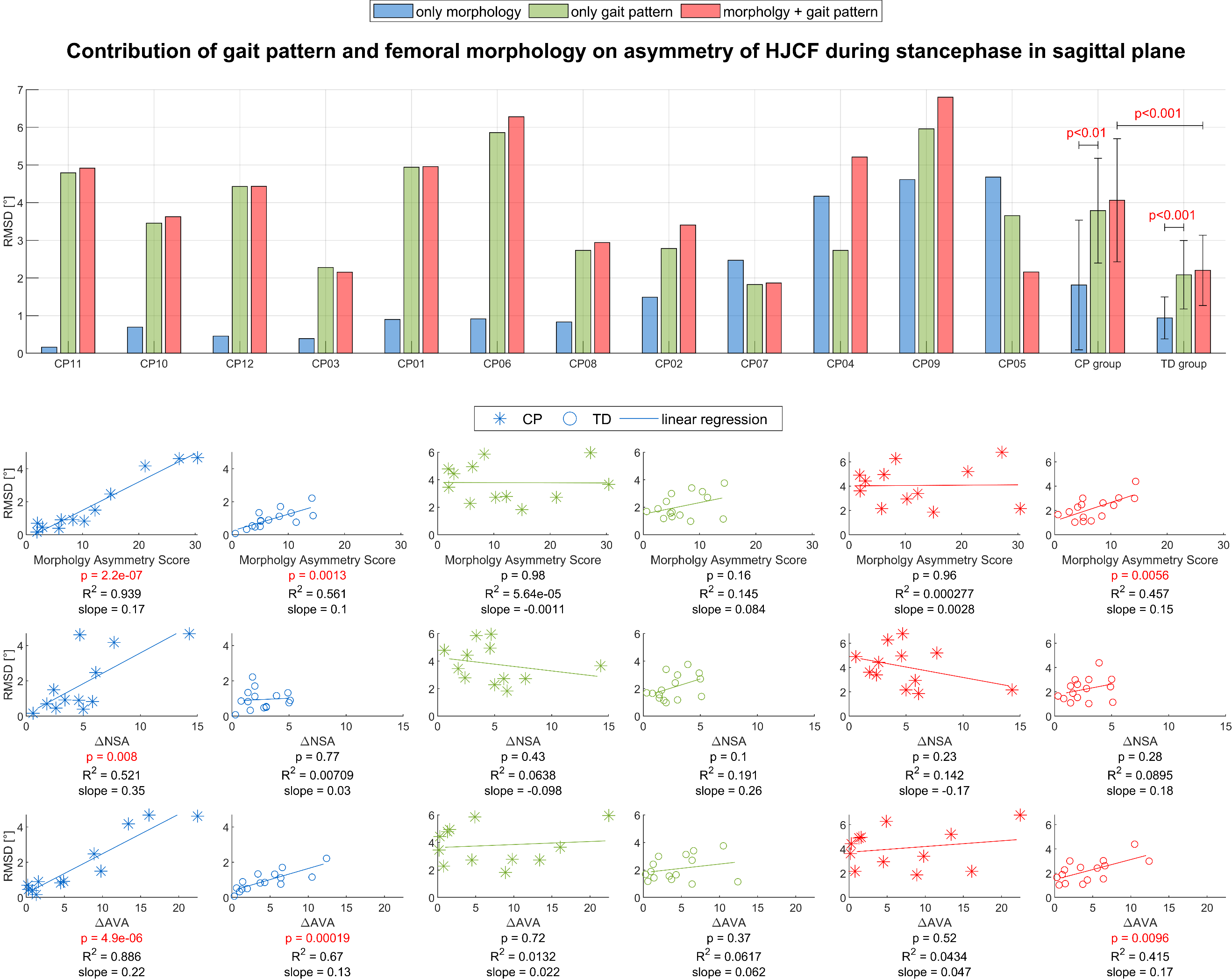


S7 Fig. RMSD between left and right HJCF orientation in sagittal plane due to the femoral morphology (blue), the child’s gait pattern (green) and both combined (red). Below scatter plots showing the correlation between asymmetric HJCF and femoral morphology
